# Minimum Dietary Diversity for Adolescents: Multicountry Analysis to Define Food Group Thresholds Predicting Micronutrient Adequacy among Girls and Boys Aged 10–19 Years

**DOI:** 10.1016/j.cdnut.2024.102097

**Published:** 2024-02-08

**Authors:** Giles Tristan Hanley-Cook, Sara Hoogerwerf, Juan Pablo Parraguez, Simone Michelle Gie, Bridget Anna Holmes

**Affiliations:** Food and Nutrition Division (ESN), FAO of the United Nations, Rome, Italy

**Keywords:** dietary surveillance, FAO/WHO GIFT, healthy diet metrics, usual intake, validation

## Abstract

**Background:**

Adolescents’ diets have been overlooked in nutrition information systems, interventions, and policies. The minimum dietary diversity for women (MDD-W) indicator has been validated to signal greater micronutrient adequacy among nonpregnant women from low- and middle-income countries, but there is limited evidence for valid food group thresholds among boys or nonpregnant nonlactating girls.

**Objective:**

To define a food group threshold that reflects minimum dietary diversity for adolescents.

**Methods:**

This multicountry study evaluated the test characteristics of a 10-point food group diversity score (FGDS)—underlying MDD-W—and food group thresholds to predict the micronutrient adequacy of diets from single 24-h recalls or food diaries (24-HRs) among 83,935 adolescents aged 10–19 y and repeated 24-HRs among 75,480 adolescents from upper-middle and high-income countries.

**Results:**

FGDS was lowest among adolescents in lower-middle countries (3.5 ± 1.1) and greatest in high-income countries (5.4 ± 1.3 points). Using single 24-HRs, 1-point increments in FGDS performed identically to predict a higher mean adequacy ratio among boys and girls (5.1 percentage points; 95% confidence interval: 5.0, 5.2; *P* < 0.001). MDD-W (i.e., ≥5 food groups) performed well in predicting a mean adequacy ratio of >0.60 among adolescents from upper-middle and high-income countries, whereas a ≥4 food group cutoff showed a superior balance between sensitivity, specificity, and percentage correctly classified in low (only girls) and lower-middle-income countries (boys and girls). In contrast, using repeated 24-HRs, the mean probability of adequacy levels among adolescents were too high and homogeneous (i.e., all mean probability of adequacies > 0.60) to define an optimal food group threshold.

**Conclusions:**

MDD-W can be extended to boys and girls aged 10–19 y from upper-middle and high-income countries. Furthermore, an adapted indicator using a ≥4 food group threshold signals higher micronutrient adequacy in low and lower-middle-income countries. Food group cutoffs to predict the micronutrient adequacy of usual intakes should be validated using repeated 24-HRs in populations where a lower proportion of adolescents meet mean dietary requirements.

## Introduction

Adequate nutrition during adolescence is crucial, as this phase of life is characterized by greater nutrient requirements because of rapid physical growth—second only to the first 1000 d from conception to 2 y—and significant physiologic, cognitive, and emotional development [1]. Therefore, adolescence is a promising entry point to foster healthy diets, as individuals gradually gain independence from their primary caregivers and subsequently develop habits, behaviors, and responsibilities related to the acquisition, preparation, and consumption of food and drinks that may have a lifelong impact on both their own health and that of future generations [2]. Nonetheless, adolescents’ diets have been largely overlooked in nutrition information systems, interventions, and policy research, reflected partly in the absence of standardized dietary indicators [3].

No single food or food group provides the multitude of nutrients and bioactive nonnutrients essential for optimal nutrition and long-term health [4]. Diverse diets, based on a wide variety of nutrient-dense foods between and within food groups, are associated with a greater likelihood of covering micronutrient requirements [[5], [6], [7]] and lower rates of noncommunicable diseases [8,9]. Hence, dietary diversity—a key component of, but not synonymous with, diet quality [10]—is a long-standing public health principle advocated in most food-based dietary guidelines [11], the WHO’s “Healthy Diet” [12] fact sheet and WHO and the FAO of the United Nations guiding principles for “Sustainable healthy diets” [10].

At present, food group diversity modules are being integrated into large-scale, multitopic data collection efforts [e.g., minimum dietary diversity for women (MDD-W) questionnaire in the Demographic and Health Surveys and Gallup World Poll], given their relatively low resource requirements, short enumeration time, and comparative simplicity (e.g., no intake quantities required) [13]. WHO has validated a dichotomous 4 out of 7 food group indicator to reflect the micronutrient density adequacy of complementary food for infants and young children (6–23.9 mo) [14,15]. However, the threshold was recently revised to 5 out of 8 food groups (including breastmilk) without additional validation against the nutrient (density) adequacy of diets [16,17]. The Women’s Dietary Diversity Project (WDDP) and its follow-up (i.e., WDDP-II) also developed and validated simple food group diversity scores as predictors of micronutrient adequacy, using data from 6 African and Asian low- and middle-income countries. More specifically, the WDDP-II validated MDD-W, a dichotomous indicator reflecting the proportion of nonpregnant women aged 15–49 y who consumed ≥5 out of 10 food groups over the previous 24-h, to signal a mean probability of adequacy (MPA) of >0.60 for 11 micronutrients.

More recently, MDD-W was shown to reflect a greater mean adequacy ratio (MAR) of 17 micronutrients among nonpregnant women from 8 Latin-American countries [18]. However, an adapted ≥6 food group cutoff markedly improved the indicator’s performance to predict an MPA > 0.60 among pregnant girls (13–19 y) and pregnant women of reproductive age in Bangladesh [19], whereas a ≥4 food group threshold showed superior test characteristics for an MPA of >0.75 among children aged 24–59 mo in Burkina Faso [20]. Furthermore, ≥4, ≥5, and ≥6 food groups were most predictive of an MPA between 0.66 and 0.80 among preschool children (1–4 y), school-aged children and adolescents (5–19 y), and adults and the elderly in Mexico (≥20 y), respectively [21]. Similarly, MDD-W (i.e., ≥5 food groups) showed the best predictive capacity for an MPA > 0.60 among children (2–9 y) in China, whereas ≥6 food groups performed more adequately among adolescents, women, men, and the elderly (≥10 y) [22]. Lastly, among adolescents (13–18 y) in Costa Rica, ≥4 food group threshold best predicted a MAR of ≥0.70 [23].

To help operationalize the measurement of dietary diversity across the life course, this study aims to assess the validity of population-level food group cutoffs for adolescent boys and nonpregnant nonlactating (NPNL) adolescent girls (10–19 y) to predict minimally acceptable levels of micronutrient adequacy across contexts (i.e., MAR or MPA > 0.60), using open access, multicountry (repeated) quantitative 24-h recall or food diary (24-HR) datasets available on the FAO/WHO Global Individual Food consumption data Tool (GIFT) platform.

## Methods

Our research is reported using the STROBE-nut checklist [24]. Furthermore, our analysis considers—as per the United Nations definition—adolescents to be individuals aged 10–19 y [25].

### Data sources

Open access data for our analyses were obtained from the FAO/WHO GIFT platform on 27 June 2022 [26].

For analyses using only the first or single day quantitative 24-HRs, we used secondary cross-sectional survey data from 438 NPNL adolescent girls from 5 low-income countries [[27], [28], [29], [30], [31], [32]], 3571 boys from 3 and 4023 NPNL girls from 7 lower-middle income counties [7,[33], [34], [35], [36], [37], [38], [39], [40], [41], [42], [43], [44]], 33,596 boys and 41,434 NPNL girls from 4 upper-middle-income countries [[45], [46], [47], [48]], and 428 and 445 boys and NPNL girls from 2 high-income countries, respectively [[49], [50], [51]]. No minimum sample size or year of data collection restrictions (range: 2003–2018) were imposed for survey inclusion, and our main findings are presented aggregated by World Bank country income classification (i.e., low, lower-middle, upper-middle, and high-income countries) [52].

For analyses using the available repeated 24-HRs, we used data from boys in Brazil (*n* = 32,059), Italy (*n* = 139), and Mexico (*n* = 1411) and NPNL girls in Brazil (*n* = 39,912), Italy (*n* = 162), Mexico (*n* = 1369), and Mozambique (*n* = 428). The minimum sample size for survey inclusion was ≥100 adolescents [53], with a repeated 24-HR conducted on a nonconsecutive day among a subsample of no <40 individuals or 10% and 5% when the sample sizes were >400 and 1000 individuals, respectively. The repeated 24-HRs were collected from a random subsample in Brazil [46], Mexico [45], and Mozambique [29] without examining the representativeness of the general adolescent population and from the entire sample in Italy [49].

### Food group diversity score and cutoffs

Low-resource healthy diet metrics have been developed specifically for situations in which simplicity during data collection and processing is a high priority and where it is not considered feasible to collect repeated 24-HRs. Hence, adolescents’ 10-point food group diversity score was constructed by summing the number of food groups consumed in ≥15 g quantities using data from the first 24-HR only [54]. The 10 predefined food groups, which underlie the validated MDD-W indicator, are as follows: *1*) starchy staple foods; *2*) pulses (beans, peas, and lentils); *3*) nuts and seeds; *4*) dairy products (milk, yogurt, and cheese); *5*) flesh foods (meat, fish and seafood, poultry, and liver or organ meats); *6*) eggs; *7*) dark green leafy vegetables; *8*) vitamin A-rich fruits and vegetables; *9*) other vegetables; and *10*) other fruits. Food group cutoffs, to be assessed for their predictive capacity of micronutrient adequacy, were defined as consuming ≥1 through a maximum of 10 food groups across the previous 24 h. As a sensitivity analysis—to reflect how MDD-W data collection is operationalized (e.g., the survey module should only include food item examples usually consumed in quantities ≥15 g/d)—adolescents’ 10-point food group diversity score was recalculated by only counting a food group as consumed when ≥1 food item was eaten in a minimum of 15 g.

### Single day 24-HR data: MAR of intakes

To estimate the micronutrient adequacy of the diet, the Nutrient Adequacy Ratios (NARs) were calculated for 11 micronutrients [18]: calcium, folate, iron, niacin, riboflavin, thiamin, vitamin A, vitamin B6, vitamin B12, vitamin C, and zinc. Vitamin A intakes were expressed in μg/d of Retinol Equivalents, whereas other micronutrient intakes were expressed as mg/d or μg/d (i.e., folate and vitamin B12). The NAR value for a given nutrient is the ratio of an individual’s nutrient intake to the Estimated Average Requirement (EAR) for the corresponding sex, age, and physiologic status category (i.e., cutpoint approach). This study employed the harmonized EARs proposed by Allen et al. [55], which are a combination of values from the United States National Academy of Medicine and the European Food Safety Authority (Supplemental Table 1). Following Passarelli et al. [56], the bioavailability of iron (i.e., low, medium, and high) and zinc (i.e., unrefined, semiunrefined, semirefined, and refined) was determined by a country’s Human Development Index (i.e., low, medium, high, and very high) (Supplemental Table 2). NARs are truncated at 1, indicating intakes are equal to or above the EAR, so that high consumption of 1 micronutrient does not compensate for low consumption of another. For each adolescent, the MAR was calculated as the sum of the NARs of available micronutrients in the respective survey, divided by the number of micronutrients. Lastly, a MAR of >0.60 was defined as the cutoff reflecting a minimally acceptable level of dietary micronutrient adequacy to ensure comparability with the multicountry analysis originally undertaken to validate MDD-W as a predictor of micronutrient adequacy among nonpregnant women aged 15–49 y [6].

### Repeated 24-HR data: MPA of usual intakes

The probability of adequacy (PA) for each micronutrient of interest was estimated through the probability approach [57], which is based on data (or assumptions) regarding the distribution of nutrient requirements in the population and the day-to-day variability of nutrient intakes, using the harmonized EARs proposed by Allen et al. [55] (Supplemental Table 1). The coefficients of variation, and consequently the back-calculated SDs, were used from the WHO and FAO [58], if available, or otherwise from the National Academy of Medicine [57]. For the day-to-day variance in micronutrient (and energy) intakes, this study leveraged the repeated 24-HR data collected within specific surveys on FAO/WHO GIFT. The ratios of intraperson to total variance were reviewed to determine whether any nutrient intakes were >0.9, which was considered a result of outliers and implausible [59]. To compute the PAs, skewed micronutrient intakes were transformed using a Box–Cox transformation. Thereafter, the individual and population mean for intakes of each micronutrient were calculated using the transformed variables to obtain symmetrical distributions. Subsequently, using the intra- and interperson variances of the transformed intake variables, the best linear unbiased predictor (BLUP) of the usual intake of each nutrient was calculated for each adolescent boy and NPNL girl [60]. Depending on the relative size of intra- to interperson variability in intake for each micronutrient, the BLUP shrinks the individual-level mean intake toward the overall group mean. When information on the distribution of requirements (coefficients of variation or SD) was available, distributions were assumed to be approximately normal. The requirement distribution for each nutrient was simulated (i.e., random normal variable; *n* = 1000) and transformed using an identical Box–Cox transformation as used for micronutrient intakes. As previously described, the bioavailability of iron and zinc was determined by a country’s Human Development Index (Supplemental Table 2) [56]. Then, for each adolescent, the PA was calculated for each micronutrient (i.e., the probability that the usual intake was above or equal to the requirement) based on the BLUPs and transformed requirement distributions (i.e., normal distribution with known EAR and SD). Next, the MPA was calculated for each adolescent as the sum of the PAs divided by the number of available micronutrients in the respective survey (i.e., 11, except in Italy, where zinc intakes were unavailable). Finally, an MPA > 0.60 was defined as the cutoff reflecting a minimally acceptable level of micronutrient adequacy [6].

### Statistical analysis

Data management and statistical analysis were conducted in Stata version 16.1 (StataCorp LLC, Texas, USA) [61]. Analyses were stratified by sex and reported for pooled samples and by World Bank country income classification or by individual survey. Descriptive data are presented as mean ± SD, median (IQR), or frequency (percentages). The intraperson variability in food group diversity score was computed using Stata’s “xtsum” command among adolescents with ≥2 24-HRs.

The performance of various food group cutoffs (e.g., ≥5) in predicting a MAR or an MPA > 0.60 was assessed using the AUC from receiver operating characteristics analysis and an informed assessment of the sensitivity, specificity, percentage correctly classified (PCC), and positive likelihood ratio. Following Martin-Prevél et al. [53], specificity was generally weighed slightly higher than sensitivity for cutoff validation, as false positive findings (e.g., an individual classified above the food group threshold with an MPA lower or equal to 0.60) were deemed less acceptable than false negatives results. Nonetheless, sensitivity, specificity, and PCC were all required to be >50% before a food group threshold was deemed valid. In addition, misclassification was further assessed using simple Cohen’s kappa statistics. Kappa scores of 0.21–0.40 indicate fair agreement; 0.41–0.60, moderate agreement; 0.61–0.80, substantial agreement; 0.81–1.00, almost perfect agreement [62].

Lastly, associations (unadjusted and energy-adjusted) between the 10-point food group diversity score and MAR or MPA were assessed using Spearman rank correlation coefficients and by fitting linear mixed effect regression models (random intercept for a survey). The normality of residuals was assessed by visual inspection of normal probability and quantile-normal plots. Two-sided *P* values of <0.05 were considered significant for all analyses.

### Ethical approval

All FAO/WHO GIFT data providers signed a License to Redistribute Contribution in which they warrant that: “The data included in any datasets have been collected and compiled in compliance with any legal or regulatory requirements as applicable to the Contributor” and “The data contained in the dataset(s) were collected in full informed consent of the data subject(s).”

## Results

### Sample characteristics

**FIGURE 1:**
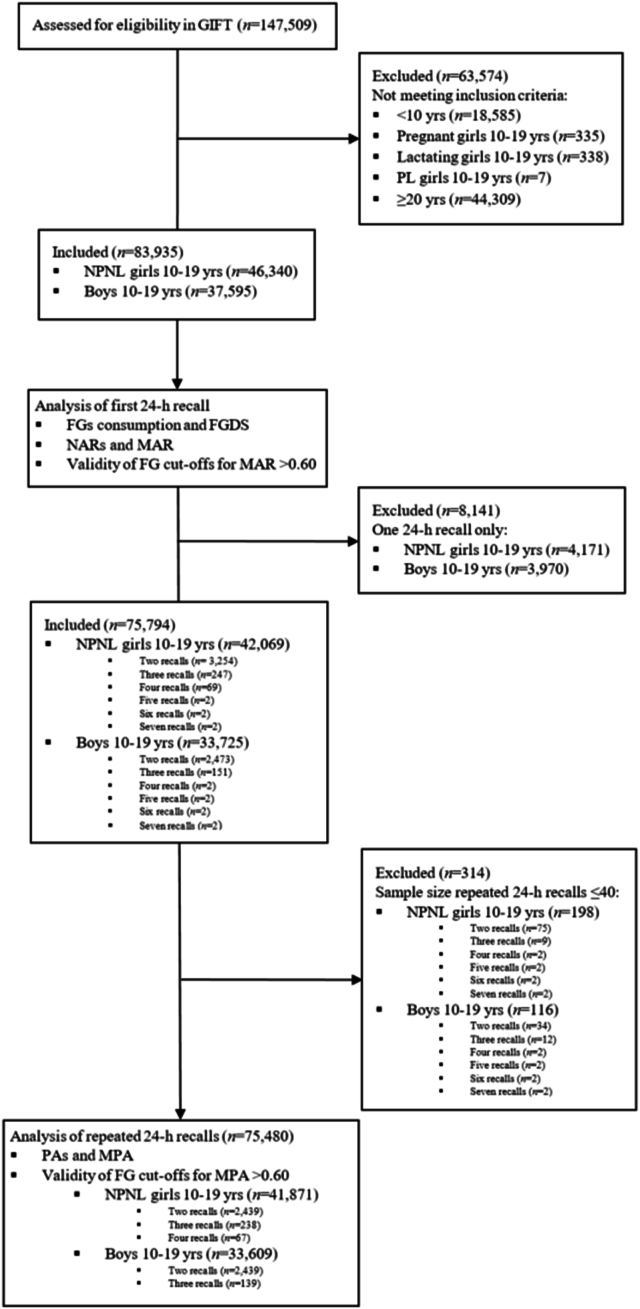
Study flowchart. FG, food group; FGDS, food group diversity score; GIFT, Global Individual Food consumption data Tool; MAR, micronutrient adequacy ratio; MPA; mean probability of adequacy; NAR, nutrient adequacy ratio; NPNL, nonpregnant nonlactating; PA, probability of adequacy; PL, pregnant and lactating.

We assessed single 24-HR data from 37,595 boys and 46,340 NPNL girls, whereas repeated 24-HR data was analyzed among 33,609 and 41,871 adolescent boys and girls, respectively (Figure 1). On mean, across both sexes, adolescents were aged 14.6 ± 1.8 y old (range: 10–19.9 y). Furthermore, food group diversity score (3.5 ± 1.1 compared with 5.4 ± 1.3 points), MDD-W (i.e., ≥5 food groups) prevalence (14.3 compared with 77.7%), MAR (0.57 ± 0.17 compared with 0.90 ± 0.13), and MAR > 0.60 prevalence (45.4 compared with 95.9%) was lowest in lower-middle and greatest in high-income countries, respectively (Table 1). Moreover, the mean intraperson variability in the food group diversity score was 0.8 food groups (range: 0.4–1.3 points) (Supplemental Tables 3 and 4).

**TABLE 1:** Sample characteristics, by World Bank income classification^1^.

	Low income^2^		Lower-middle income^3^		Upper-middle income^4^		High income^5^	
	Boys	Girls (*n* = 438)	Boys (*n* = 3571)	Girls (*n* = 4027)	Boys (*n* = 33,596)	Girls (*n* = 41,434)	Boys (*n* = 428)	Girls (*n* = 445)
Age (y)	—	16 ± 1.2 16 (15, 17)	14 ± 2.8 14 (12, 17)	14 ± 2.8 14 (12, 17)	15 ± 1.7 15 (13, 16)	15 ± 1.7 15 (13, 16)	14 ± 2.7 14 (12, 17)	14 ± 2.7 14 (12, 16)
FGDS (0–10 points)	—	3.8 ± 1.2 4 (3, 5)	3.5 ± 1.1 3 (3, 4)	3.4 ± 1.1 3 (3, 4)	4.8 ± 1.2 5 (4, 6)	4.5 ± 1.3 5 (4, 5)	5.5 ± 1.2 6 (5, 6)	5.4 ± 1.3 6 (5, 6)
MDD-W, *n* (%)	—	118 (26.9)	542 (15.2)	546 (13.6)	20,152 (60.0)	21,731 (52.5)	340 (79.4)	338 (76.0)
MAR	—	0.59 ± 0.19 0.60 (0.45, 0.75)	0.57 ± 0.17 0.58 (0.46, 0.69)	0.56 ± 0.17 0.58 (0.44, 0.69)	0.69 ± 0.19 0.72 (0.57, 0.83)	0.65 ± 0.20 0.67 (0.52, 0.81)	0.91 ± 0.11 0.95 (0.88, 0.99)	0.89 ± 0.15 0.94 (0.85, 0.99)
>0.60, *n* (%)	—	221 (50.5)	1642 (46.0)	1806 (44.9)	23,871 (71.1)	25,923 (65.6)	415 (97.0)	422 (94.8)

Abbreviations: FGDS, food group diversity score; MAR, mean adequacy ratio; MDD-W, dietary diversity for women.

1 Data are mean ± SD and median (IQR) unless otherwise stated.

2 Burkina Faso, Ethiopia, Mozambique, Uganda, and Zambia.

3 Bangladesh, Bolivia, India, Kenya, Lao People’s Democratic Republic, Nigeria, and Tanzania.

4 Argentina, Brazil, Bulgaria, and Mexico.

5 Italy and Romania.

### Associations between food group diversity score and MAR

**FIGURE 2:**
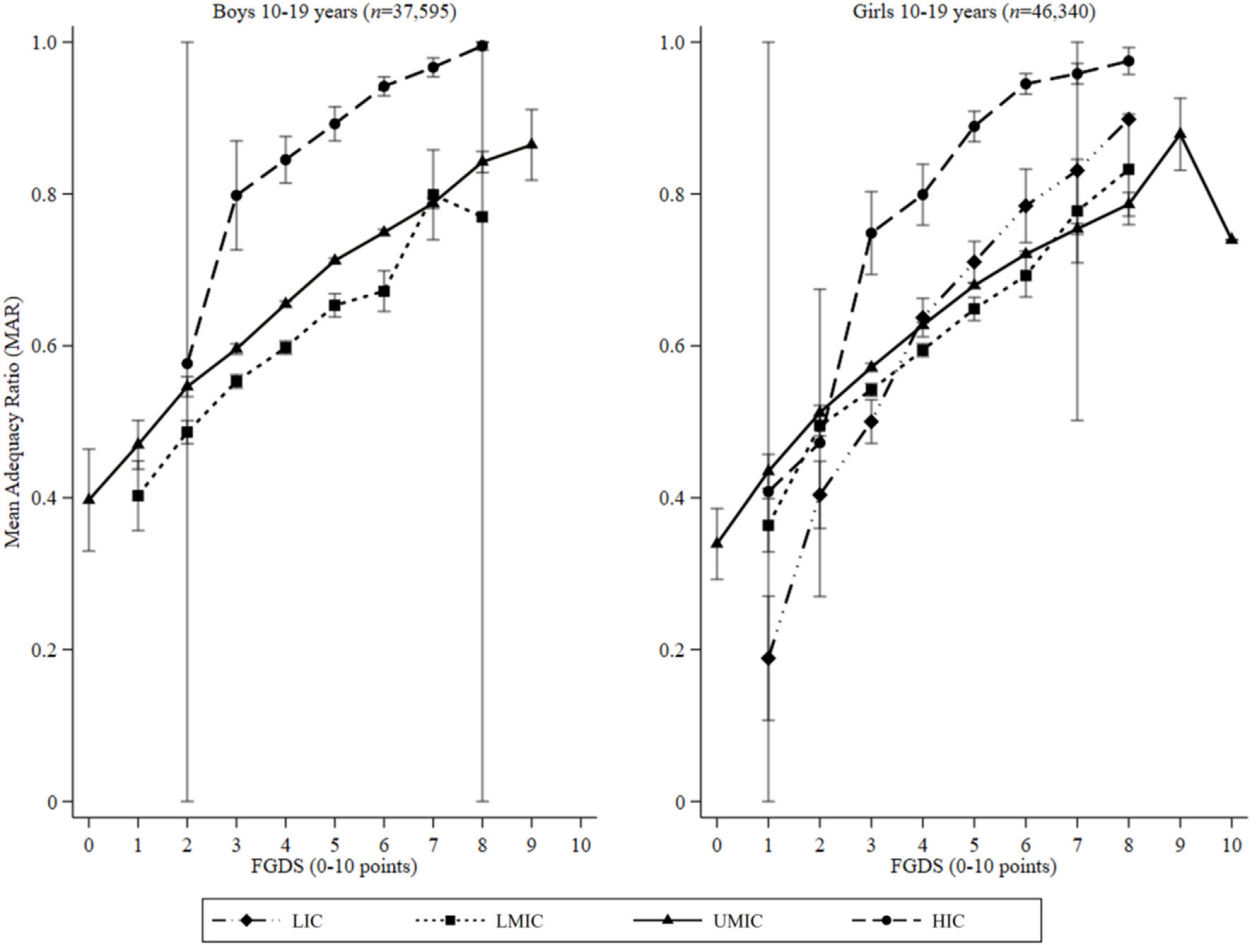
Relationships between the mean adequacy ratio and food group diversity score (FGDS) among adolescents (10–19 y). Error bars represent the 95% confidence intervals around the means. HIC, high-income country; LIC, low-income country; LMIC, lower-middle-income country; UMIC, upper-middle-income country.

The normality assumptions of residuals were met; hence, untransformed MAR data were modeled. Overall, among both boys and NPNL girls, the stratified unadjusted models indicated that 1-point increments in food group diversity score were associated with 5.1 percentage points (pp) [95% confidence interval (CI): 5.0–5.2; *P* < 0.001] higher MAR. Our findings were similar across county income classification for boys. However, among NPNL girls, associations were slightly stronger in low (10.4 pp; 95% CI: 9.2, 11.6; *P* < 0.001) and high-income countries (6.8 pp; 95% CI: 5.9–7.6; *P* < 0.001) (Figure 2; Supplemental Figures 1–5). In general, relationships between the food group diversity score and MAR were attenuated when adjusting for total energy intake (kcal/d) among boys (3.2 pp; 95% CI: 3.1, 3.3; *P* < 0.001) and NPNL girls (3.4 pp; 95% CI: 3.3, 3.5; *P* < 0.001). Moreover, the mean Spearman rank correlations were 0.36 and 0.34 among adolescent boys and girls, respectively.

### Test characteristics and preferred food group cutoffs for MAR > 0.60

**FIGURE 3:**
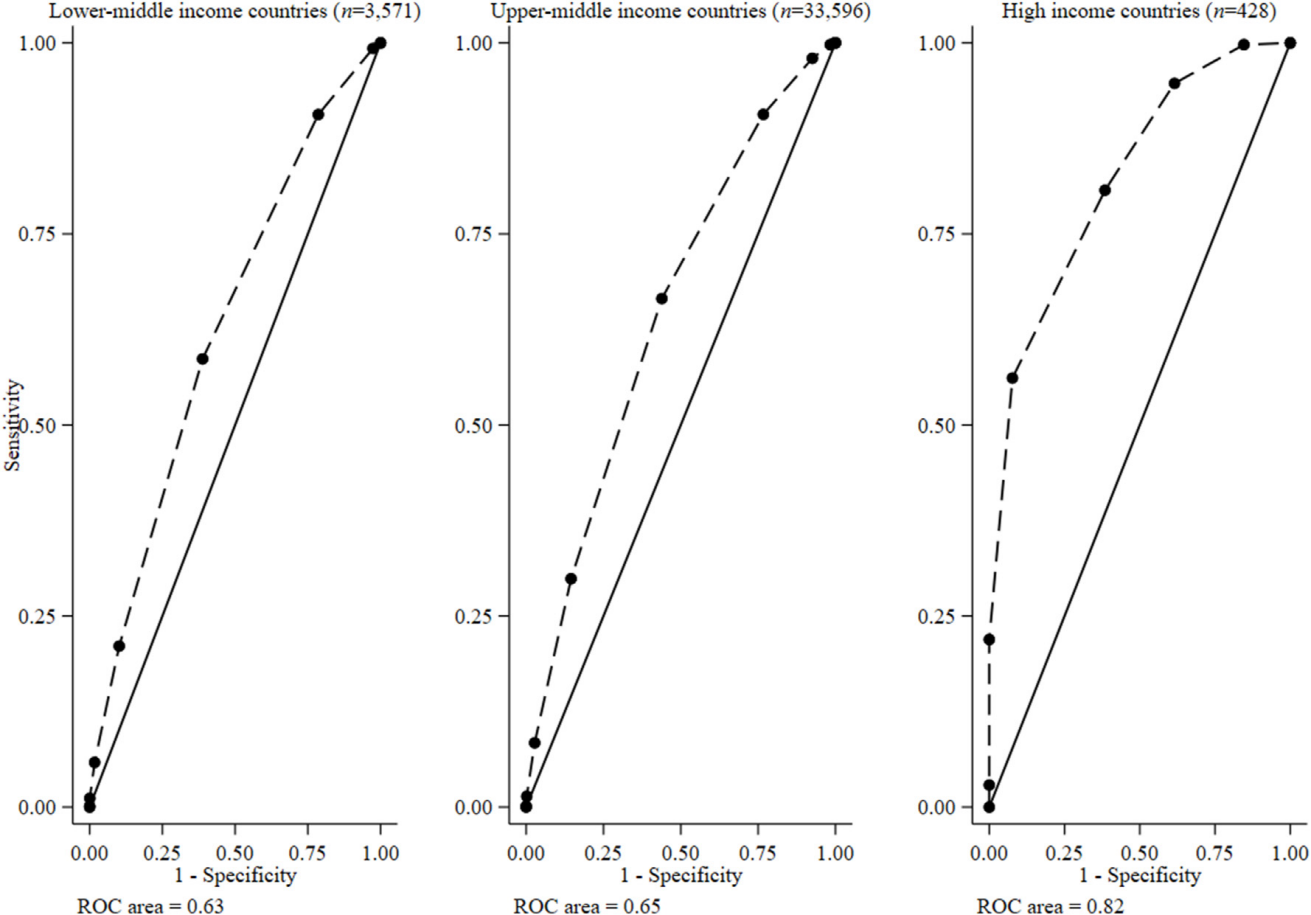
Receiver operating characteristic (ROC) curves of the food group diversity score (0–10 points) underlying MDD-W indicating predictions for adolescent boys (10–19 y) MAR > 0.60, by World Bank country income classification. The sample did not include any adolescent boys from low-income countries. MAR, mean adequacy ratio; MDD-W, minimum dietary diversity for women.

**FIGURE 4:**
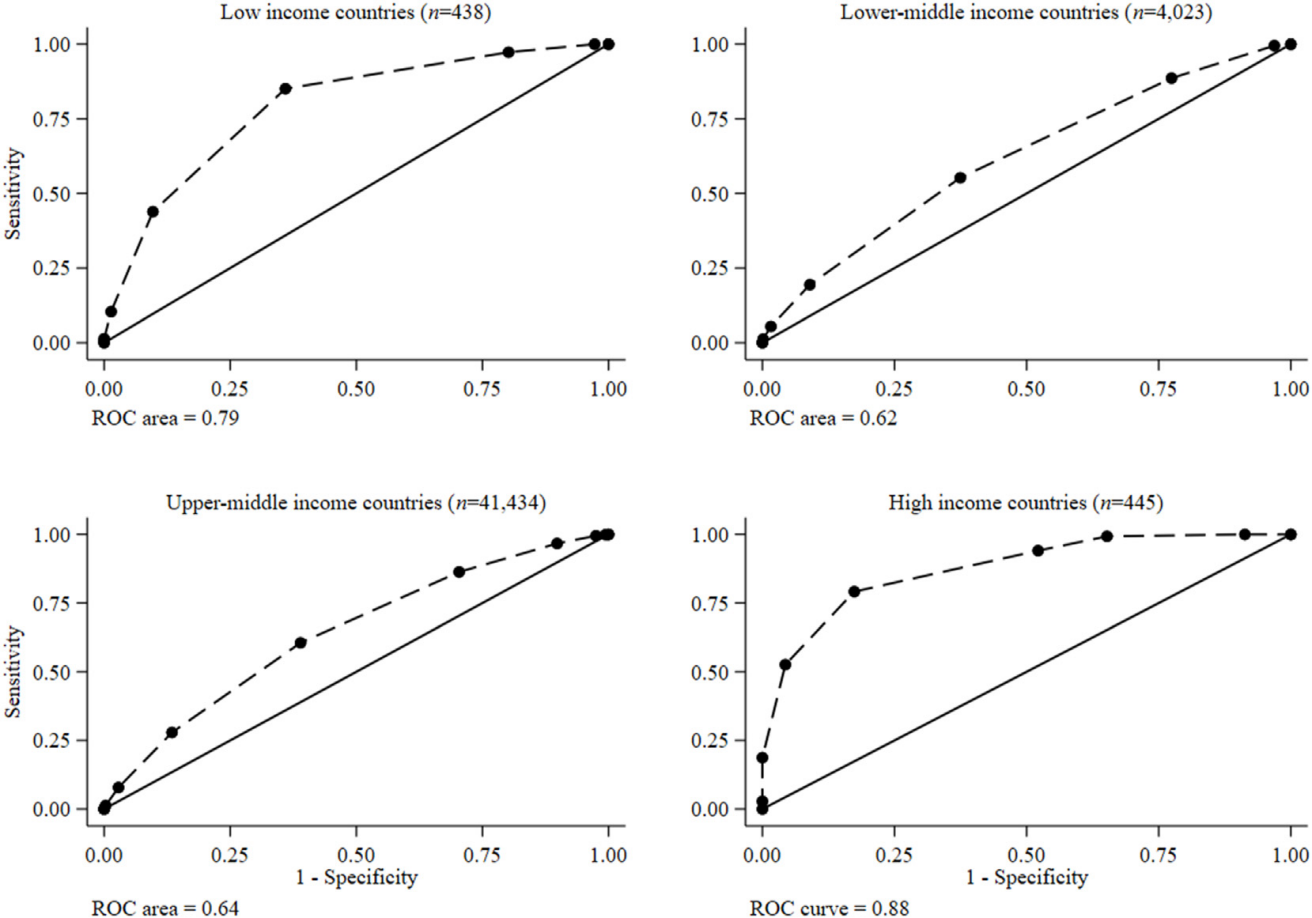
Receiver operating characteristic (ROC) curves of the food group diversity score (0–10 points) underlying MDD-W indicating predictions for nonpregnant nonlactating adolescent girls (10–19 y) MAR > 0.60, by World Bank country income classification. MAR, mean adequacy ratio; MDD-W, minimum dietary diversity for women.

Among adolescent boys from lower-middle, upper-middle, and high-income countries consuming ≥4, ≥5, and ≥5 food groups had 58.7, 66.5, and 80.7% sensitivity, 61.2, 56.1, and 61.5% specificity, and 60.0, 63.5, 80.1% PCC, and kappa scores of 0.20, 0.20, and 0.22 for a MAR > 0.60, respectively (Table 2 and Figure 3; Supplemental Tables 5 and 6, Supplemental Figure 6). Moreover, among girls aged 10–19 y from low, lower-middle, upper-middle, and high-income countries, consuming ≥4, ≥4, ≥5, and ≥5 food groups had 84.7%, 55.3%, 60.5%, and 79.2% sensitivity, 64.1%, 62.6%, 61.1%, and 82.6% specificity, 74.5%, 59.3%, 60.7%, and 79.3% PCC, and kappa scores of 0.49, 0.21, 0.20, and 0.33 for a MAR > 0.60, respectively (Table 3 and Figure 4; Supplemental Tables 7 and 8, Supplemental Figures 7 and 8). Sensitivity analyses, using single food items rather than composite food groups to “count” consumption prevalence (i.e., ≥15 g/d), confirmed our main findings (data not shown).

**TABLE 2:** Accuracy of food group cutoffs for a mean adequacy ratio > 0.6 among adolescent boys (10–19 y), by World Bank income classification^1^

Country classification	Lower-middle-income (*n* = 3571)					Upper-middle-income (*n* = 33,596)					High income (*n* = 428)				
Food group cutoff	Sensitivity	Specificity	PCC	LR+	LR−	Sensitivity	Specificity	PCC	LR+	LR−	Sensitivity	Specificity	PCC	LR+	LR−
≥1	100	0.00	46.0	1.00	—	100	0.37	71.1	1.00	0.15	—	—	—	—	—
≥2	99.3	2.59	47.1	1.02	0.28	99.6	1.66	71.4	1.01	0.15	100	0.00	97.0	1.00	—
≥3	90.6	21.4	53.2	1.15	0.44	98.0	7.47	71.8	1.06	—	99.8	15.4	97.2	1.18	0.02
≥4	58.7	61.2	60.0	1.51	0.68	90.6	23.3	71.2	1.18	0.40	94.7	38.5	93.0	1.54	0.14
≥5	21.1	89.8	58.2	2.07	0.88	66.5	56.1	63.5	1.52	0.60	80.7	61.5	80.1	2.10	0.31
≥6	5.85	98.3	55.8	3.42	0.96	29.9	85.5	46.0	2.06	0.82	56.1	92.3	57.2	7.30	0.48
≥7	1.16	100	54.5	22.3	0.99	8.40	97.2	34.1	3.04	0.94	21.9	100	24.3	—	0.78
≥8	0.12	100	54.1	—	1.00	1.41	99.8	29.9	7.23	0.99	2.89	100	5.84	—	0.97
≥9	0.00	100	54.1	1.00	—	0.12	100	29.1	11.4	1.00	0.00	100	3.04	—	1.00
>9	—	—	—	—	—	0.00	100	29.0	—	1.00	—	—	—	—	—
AUC	0.63 (95% CI: 0.61, 0.64)					0.65 (95% CI: 0.64, 0.65)					0.80 (95% CI: 0.76, 0.84)				

Abbreviations: CI, confidence interval; LR−, negative likelihood ratio; LR+, positive likelihood ratio; PCC, percentage correctly classified.

1 Values are percentages (except for the AUC values).

**TABLE 3:** Accuracy of food group cutoffs for a mean adequacy ratio > 0.6 among nonpregnant nonlactating adolescent girls (10–19 y), by World Bank income classification^1^

Country classification	Low income (*n* = 438)					Lower-middle-income (*n* = 4027)					Upper-middle-income (*n* = 41,434)					High income (*n* = 445)				
Food group cutoff	Sensitivity	Specificity	PCC	LR+	LR−	Sensitivity	Specificity	PCC	LR+	LR−	Sensitivity	Specificity	PCC	LR+	LR−	Sensitivity	Specificity	PCC	LR+	LR−
≥1	100	0.00	50.6	1.00	—	100	0.00	45.0	1.00	—	99.9	0.57	62.7	1.00	0.15	100	0.00	94.8	1.00	—
≥2	100	2.76	51.9	1.03	0.00	99.5	3.11	46.5	1.03	0.16	99.5	2.50	63.2	1.02	0.20	100	8.70	95.3	1.10	0.00
≥3	97.3	19.8	59.0	1.21	0.14	88.6	22.6	52.3	1.14	0.51	96.7	10.2	64.3	1.07	0.33	99.3	34.8	96.0	1.52	0.02
≥4	84.7	64.1	74.5	2.36	0.24	55.3	62.6	59.3	1.48	0.71	86.3	29.6	65.1	1.23	0.46	94.1	47.8	91.7	1.80	0.13
≥5	43.7	90.3	66.7	4.52	0.62	19.3	91.0	58.8	2.15	0.89	60.5	61.1	60.7	1.55	0.65	79.2	82.6	79.3	4.55	0.25
≥6	10.4	98.6	54.0	7.49	0.91	5.24	98.4	56.5	3.23	0.96	27.9	86.5	49.9	2.07	0.83	52.6	95.7	54.8	12.1	0.50
≥7	1.35	100	50.1	—	0.99	1.16	99.9	55.5	8.56	0.99	7.86	97.2	41.3	2.76	0.95	18.7	100	22.9	—	0.81
≥8	0.45	100	49.7	—	1.00	0.17	100	55.1	—	1.00	1.25	99.7	38.1	4.39	0.99	2.84	100	7.87	—	0.97
≥9	0.00	100	49.4	—	1.00	0.00	100	55.0	—	1.00	0.07	100	37.5	—	1.00	0.00	100	5.17	—	1.00
>9	—	—	—	—	—	—	—	—	—	—	0.00	100	37.5	—	1.00	—	—	—	—	—
AUC	0.63 (95% CI: 0.61, 0.64)					0.65 (95% CI: 0.64, 0.65)					0.80 (95% CI: 0.76, 0.84)					0.80 (95% CI: 0.76, 0.84)				

Abbreviations: CI, confidence interval; LR−, negative likelihood ratio; LR+, positive likelihood ratio; PCC, percentage correctly classified.

1 Values are percentages (except for the AUC values).

### Associations between food group diversity score and MPA

**FIGURE 5:**
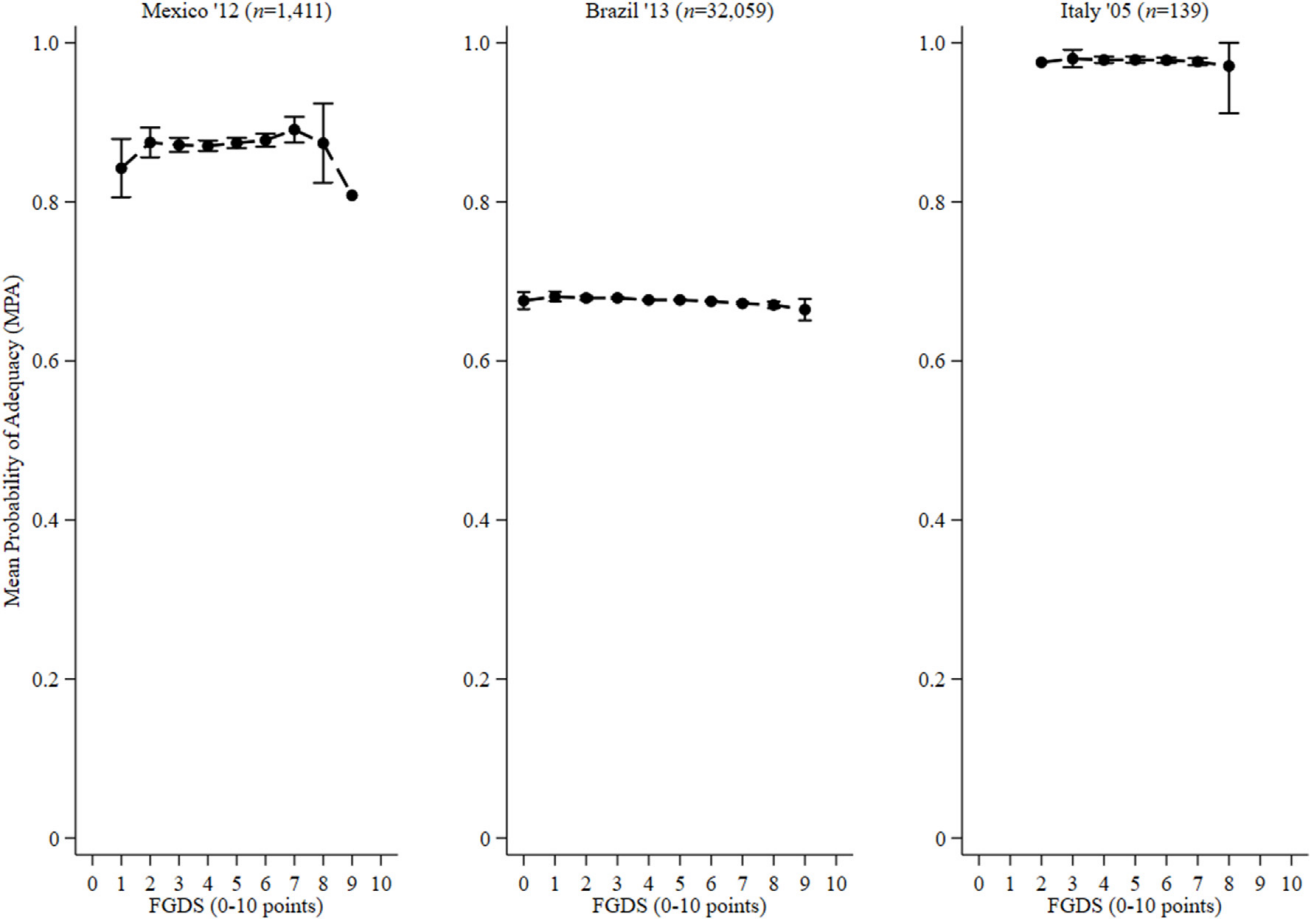
Relationships between the mean probability of adequacy of 11 micronutrients and food group diversity score (FGDS) among adolescent boys (10–19 y) from Mexico, Brazil, and Italy. Error bars represent the 95% confidence intervals around the means. Mean probability of adequacy was calculated for 10 micronutrients in Italy (zinc missing). In Mexico and Brazil, a second 24-h recall was enumerated on a nonconsecutive day among 121 and 2179 boys, whereas in Italy, second and third 24-h recalls were enumerated on a nonconsecutive day among 139 boys, respectively.

**FIGURE 6:**
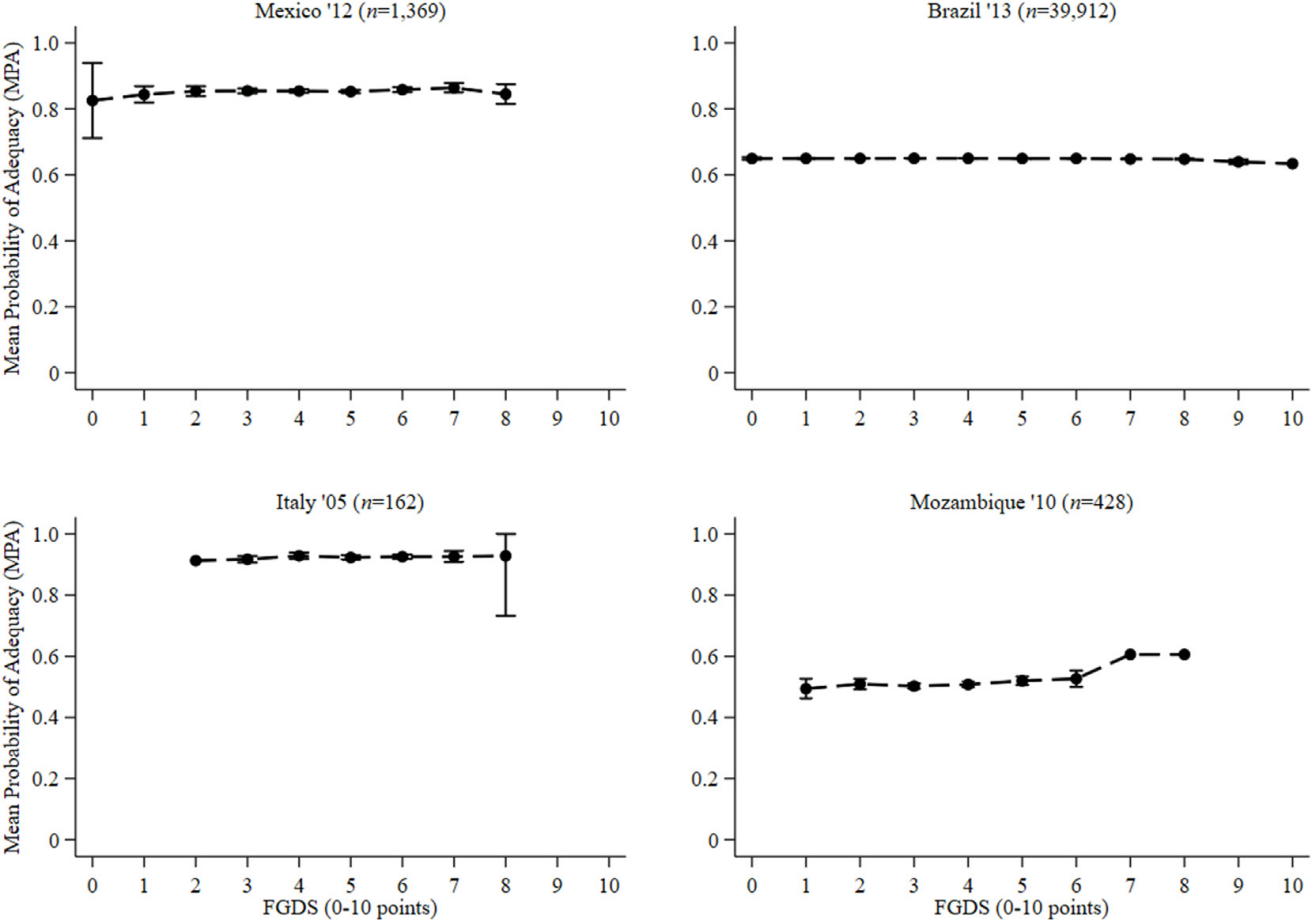
Relationships between the mean probability of adequacy of 11 micronutrients and food group diversity score (FGDS) among nonpregnant nonlactating adolescent girls (10–19 y) from Mexico, Brazil, Italy, and Mozambique. Error bars represent the 95% confidence intervals around the means. Mean probability of adequacy was calculated for 10 micronutrients in Italy (zinc missing). In Mexico and Brazil, a second 24-h recall was enumerated on a nonconsecutive day among 123 and 2807 girls, respectively. In Italy second and third 24-h recalls were enumerated on a nonconsecutive day among 139 girls. In Mozambique, second, third, and fourth 24-h recalls were enumerated on a nonconsecutive day among 87, 76, and 67 girls, respectively.

Although the normality assumptions of residuals were not met, untransformed MPA data were used as the dependent variable in regression analyses. This was because log, cubic, square, inverse, inverse square, and inverse cubic transformations failed to improve the model fit. Among adolescent boys, unadjusted analyses indicated 1 point higher food group diversity scores were associated with −0.1 pp (95% CI: −0.2, 0.0; *P* < 0.001) and nonsignificant −0.1 pp (95% CI: −0.2, 0.1; *P* = 0.37) lower MPAs in Brazil and Italy, and 0.3 pp (95% CI: 0.0, 0.6; *P* = 0.026) increments in MPA in Mexico, respectively (Figure 5). Furthermore, among NPNL girls, unadjusted analyses indicated that 1 point greater food group diversity score had null associations with MPA in Brazil (95% CI: 0.0, 0.0; *P* < 0.001) and Italy (95% CI: −0.3, 0.4; *P* = 0.77) and nonsignificant 0.1 pp (95% CI: −0.1, 0.4; *P* = 0.21) and 0.7 pp (95% CI: 0.2, 1.1; *P* = 0.005) increments in MPA in and Mexico and Mozambique, respectively (Figure 6). In addition, on mean, the Spearman rank correlations were −0.03 and 0.36 among adolescent boys and girls, respectively.

### Test characteristics and preferred food group cutoffs for MPA > 0.60

**FIGURE 7:**
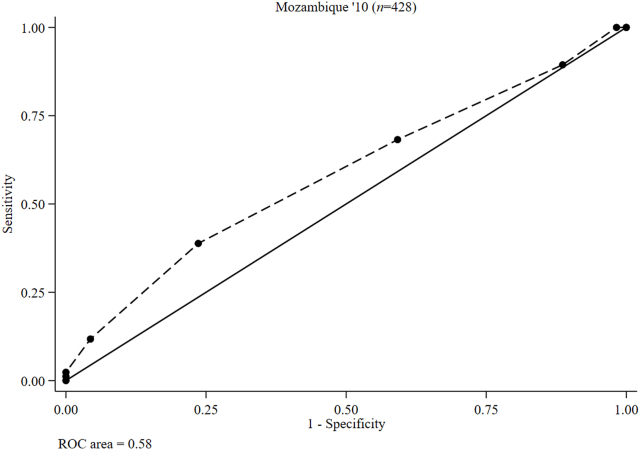
Receiver operating characteristic (ROC) curves of the food group diversity score (0–10 points) underlying MDD-W indicating predictions for nonpregnant nonlactating adolescent girls (10–19 y) mean probability of adequacy > 0.60 for 11 micronutrients in Mozambique. MDD-W, minimum dietary diversity for women.

Because of a lack of heterogeneity in MPA among boys aged 10–19 y, no food group cutoff could be defined to predict an MPA of >0.60. To clarify, the usual micronutrient intakes of all adolescent boys from Brazil, Italy, and Mexico had a composite MPA of >0.60 (Supplemental Table 9). For identical reasons, no food group cutoff could be defined to predict an MPA of >0.60 among NPNL girls from Brazil, Italy, and Mexico. However, in Mozambique, only 19.9% of girls achieved an MPA of >0.60, and consuming ≥5 food groups had a 38.8% sensitivity and 76.4% specificity for predicting an MPA of >0.60 (Table 4 and Figure 7; Supplemental Table 10).

**TABLE 4:** Accuracy of food group cutoffs for a mean probability of adequacy > 0.6 among nonpregnant nonlactating adolescent girls (10–19 y) from Mozambique^1^

Food group cutoff	Mozambique ‘10 (*n* = 428)				
	Sensitivity	Specificity	PCC	LR+	LR−
≥1	100	0.00	19.9	1.00	—
≥2	100	1.75	21.3	1.02	0.00
≥3	89.4	11.4	26.9	1.01	0.93
≥4	68.2	40.8	46.3	1.15	0.78
≥5	38.8	76.4	68.9	1.64	0.80
≥6	11.8	95.6	79.0	2.69	0.92
≥7	2.35	100	80.6	—	0.98
≥8	1.18	100	80.4	—	0.99
≥9	0.00	100	80.1	—	1.00
>9	—	—	—	—	—
AUC	0.58 (95% CI: 0.51, 0.65)				

Abbreviations: CI, confidence interval; LR−, negative likelihood ratio; LR+, positive likelihood ratio; PCC, percentage correctly classified.

1 Values are percentages (except for the AUC values).

## Discussion

In this study, we determined food group thresholds for the MDD-W indicator to predict a MAR larger than 0.60 among adolescent boys and NPNL girls across World Bank country income classifications. Leveraging over 80,000 single quantitative 24-HRs, the conventional ≥5 food group MDD-W cutoff showed the optimal balance between sensitivity, specificity, and PCC among boys and girls aged 10–19 y from upper-middle (i.e., Brazil and Mexico) and high-income countries (i.e., Italy and Romania). Furthermore, a 4 or higher food group threshold showed superior test characteristics among NPNL girls in low-income countries (i.e., Mozambique) and across both sexes in lower-middle income countries (i.e., India and Kenya). In contrast, when using repeated 24-HRs to model usual micronutrient intakes, our analyses were unable to validate an optimum food group cutoff, as all adolescents from Brazil, Italy, and Mexico had an MPA of >0.60 and the predictive capacity of food group thresholds for micronutrient adequacy were poor in Mozambique.

The heterogeneous food group thresholds for defining a dichotomous indicator of acceptable dietary micronutrient adequacy are unsurprising, given different age ranges, physiological statuses, and dietary patterns (e.g., using the Human Development Index as a proxy for iron and zinc bioavailability [56]) are associated with distinct EARs [55]. To illustrate, Nguyen et al. [19] reported that using the MDD-W indicator’s ≥5 food group threshold led to a high percentage of misclassification among pregnant adolescents aged 13–19 y and pregnant women—whose requirements are higher than nonpregnant individuals—and therefore recommended a ≥6 food group cutoff to predict an MPA > 0.60. The optimal cutoffs of ≥4 and ≥5 for MAR > 0.60 for adolescents defined in this study are consistent with this expectation, as boys and NPNL girls aged 10–19 y have similar or marginally higher requirements to nonpregnant women—relative to their energy requirements—and lower requirements than pregnant or lactating women [55,63].

Furthermore, our findings using single 24-HRs in Mexico indicated that a ≥5 food group cutoff best predicted a MAR > 0.60 among boys and NPNL girls aged 10–19 y. These results match previous research among adolescents aged 10–14, 15–19 [22], and 12–19 y in Mexico [21]. Rodríguez-Ramírez et al. [21] reported that their ≥5 food group threshold had a sensitivity of 70.1% and a specificity of 59.4% to predict an MPA > 0.66, which better aligns with the test characteristics of the ≥4 food group cutoff in our analysis. In addition, our estimated mean MAR and MPA of ∼0.80 and ∼0.85, respectively, among Mexican boys and NPNL girls were similar to a recent study reporting a mean MPA of ∼0.70 among adolescents aged 12–19 y [21] but was substantially higher than the mean MPAs of ∼0.45 and ∼0.30 among boys and girls aged 10–14 and 15–19 y, respectively [22]. Moreover, in contrast to Rodríguez-Ramírez et al. [21] and Arimond et al. [22], our study followed the minimal acceptable level of micronutrient adequacy defined during MDD-W validation among nonpregnant women [6] and consequently did not assess the test characteristics of food group cutoffs for other binary MAR or MPA thresholds (e.g., larger than 0.70 or 0.80).

Our study also indicated that MDD-W prevalence (i.e., ≥5 food groups) ranged between <15% in India to >70% in Italy and Romania among boys and NPNL girls. For the pooled sample, the consumption prevalence of nuts and seeds, eggs, and dark green leafy vegetables was <20%. Furthermore, intakes of calcium, vitamin A, vitamin B6, and vitamin C were least likely to cover EARs (<60%) across both sexes. Our study found that <35% of adolescents in Lao People’s Democratic Republic achieved a MAR > 0.60, whereas the adequacy cutoff was reached by ≥80% in Mexico, Bolivia, Argentina, Italy, Romania, Bulgaria, and Nigeria. Furthermore, our results showed that the food group diversity score was positively associated and correlated with MAR among adolescents. These findings are consistent with the established body of literature indicating that dietary diversity is strongly related to dietary micronutrient adequacy (and energy intakes) of children [20,64] and women at various physiologic statuses in low- and middle income countries [6,19].

Our usual intake analyses were unable to determine valid food group cutoffs to predict an MPA > 0.60 among adolescents. In summary, because of the large intraperson variance in micronutrient intakes, no boys or girls aged 10–19 y had an MPA ≤ 0.60. Overall, only calcium, vitamin A, vitamin C, and vitamin C had PAs of <95%. These findings—which are in contrast to the main results using MAR > 0.60—can be explained by regression to the relatively higher means in upper-middle (i.e., Brazil and Mexico) and in high-income countries (i.e., Italy) [65]. To clarify, individuals reporting inadequate micronutrient intakes (and low dietary diversity) during the first 24-HR were, on mean, more likely to have more adequate intakes on the subsequent nonconsecutive day(s) of the repeated 24-HR. Among NPNL girls from Mozambique, this phenomenon was slightly less apparent; however, no food group threshold met our prespecified test characteristics criteria of sensitivity, specificity, and PCC, all being ≥50%.

Our study has several limitations. First, quantitative 24-HR data on FAO/WHO GIFT are heterogeneous in terms of micronutrient coverage, year and season of data collection, sample sizes and weighting (e.g., urban and rural) of vulnerable population groups, national representativeness, the 24-HR methodology employed (e.g., multiple passes and real-time diary), and prevalence of over- and underreporting. Hence, our pooled estimates—presented for didactic simplicity—should be interpreted with caution (e.g., Brazil accounts for >90% of the sample size of upper-middle-income countries). Second, no data were available from boys in low-income countries, whereas the sample sizes of adolescents from low and high-income countries were limited for both sexes (i.e., <500 individuals). Third, single 24-HRs are prone to random error because of the natural day-to-day variability in nutrient intake. Hence, all healthy diet metrics based on a single 24-HR lack precision [4]. Furthermore, using the cutpoint approach to compute MAR likely leads to an overestimation of inadequacy (i.e., by inflating the lower tail of the intake distribution [66]). Hence, additional analyses of datasets with repeated 24-HRs across geographic settings, with lower proportions of adolescents meeting mean dietary requirements, are strongly recommended to confirm our main findings using single 24-HRs. Fourth, dietary diversity cannot be interpreted as overall diet quality, which also requires macronutrient adequacy and balance and moderation of unhealthy foods and nutrients to limit (e.g., sodium, free sugars). Fifth, no standalone MDD-W questionnaire was used to measure food group consumption, resulting in the best-case scenario to discover relationships between the food group diversity score and MAR or MPA. To clarify, in most studies, the prevalence of food group consumption is assessed using simpler methods like open qualitative 24-HRs (i.e., without portion size estimation) or list-based questionnaires (i.e., asking a respondent if food groups were consumed or not) [54]. Previous research has indicated that simpler and more scalable data collection methods to count food groups result in overreporting of food group consumption, compared with quantitative dietary assessments, which were considered reference methods [13,67,68]. Nonetheless, our study also has several important strengths. First, all datasets on FAO/WHO were harmonized with the European Food Safety Authority’s FoodEx2 food classification and description system. This harmonization was aimed at enhancing the consistency and reliability of nutrient intake and dietary exposure assessments across contexts [26]. Second, our multicountry analyses covered a large sample of adolescents’ diets across all 4 World Bank country income classifications, owing to the harmonized data shared through FAO/WHO GIFT. Third, nonconsecutive day 24-HRs were available in a subsample of boys and NPNL girls from 3 and 4 countries, respectively. These data allowed us to account for the day-to-day variance in micronutrient intakes and were used to estimate the PAs for 11 micronutrients, taking into account the distribution of requirements.

In conclusion, MDD-W can be extended to boys and NPNL girls aged 10–19 y from upper-middle and high-income countries (i.e., for relative ranking). Furthermore, an adapted indicator using a ≥4 food group threshold signals higher micronutrient adequacy in low and lower-middle income countries. Nonetheless, we acknowledge that from a global MDD-W guidance perspective [54], it would be more convenient to recommend a uniform cutoff of ≥5 food groups for adolescents across contexts to align with its use among women of reproductive age. Moreover, expanding the existing, simple, and widely collected MDD-W indicator to adolescents represents an opportunity to increase dietary data collection among this historically underserved group. Future research should replicate our study among boys and girls in other settings (e.g., urban, periurban, and rural). In addition, MDD-W validation efforts should be extended to other population groups worldwide (e.g., pregnant women, men, and elderly).

## Author contributions

The authors’ responsibilities were as follows – The authors’ responsibilities were as follows: GTH-C, BAH: designed research; GTH-C: analyzed data and performed statistical analysis; GTH-C, SH, SMG: wrote the paper; GTH-C, BAH: have primary responsibility for final content; and all authors: read and approved the final manuscript.

## Conflict of interest

The authors report no conflicts of interest.

## Funding

This work was supported by the German Development Cooperation Agency (GIZ)—Knowledge for Nutrition (K4N). The funder had no role in the study design, data analysis, decision to publish, or preparation of the manuscript.

## Data availability

All data supporting the reported results are publicly available on the FAO/WHO Global Individual Food consumption data Tool (FAO/WHO GIFT) website: https://www.fao.org/gift-individual-food-consumption/data/en.

## Disclaimer

The views expressed in this publication are those of the authors and do not necessarily reflect the views or policies of the FAO of the United Nations.

## Appendix A Supplementary data

Multimedia component1 Multimedia component1
