# Supplementary material for: Minimum Dietary Diversity for Adolescents: Multicountry Analysis to Define Food Group Thresholds Predicting Micronutrient Adequacy among Girls and Boys Aged 10–19 Years
Source: Curr Dev Nutr. 2024 Feb 8;8(3):102097. doi: 10.1016/j.cdnut.2024.102097 (PMC10899069; doi:10.1016/j.cdnut.2024.102097)
Supplement: Multimedia component1 [file mmc1.docx]

**Supplemental table 1. Estimated Average Requirements (EARs) and standard deviations (SD) used to assess micronutrient adequacy among adolescent boys and non-pregnant non-lactating girls aged 10-19 years^1^**

|  | **Male**  **(7-10 years)** | | **Female**  **(7-10 years)** | | **Male**  **(11-14 years)** | | **Female**  **(11-14 years)** | | **Male**  **(15-17 years)** | | **Female**  **(15-17 years)** | | **Male**  **(18-24 years)** | | **Female**  **(18-24 years)** | |
| --- | --- | --- | --- | --- | --- | --- | --- | --- | --- | --- | --- | --- | --- | --- | --- | --- |
|  | **EAR** | **SD** | **EAR** | **SD** | **EAR** | **SD** | **EAR** | **SD** | **EAR** | **SD** | **EAR** | **SD** | **EAR** | **SD** | **EAR** | **SD** |
| Calcium (mg/day)^2^ | 680 | 85 | 680 | 85 | 960 | 120 | 960 | 120 | 960 | 120 | 960 | 120 | 860 | 108 | 860 | 108 |
| Folate (µg/day)^3^ | 160 | 16 | 160 | 16 | 210 | 21 | 210 | 21 | 250 | 25 | 250 | 25 | 250 | 25 | 250 | 25 |
| Iron (mg/day) 16% absorption (high)^3^ | NA | NA | NA | NA | 8 | 0.8 | 7 | 0.7 | 8 | 0.8 | 7 | 0.7 | 6 | 0.6 | 7 | 0.7 |
| Iron (mg/day) 10% absorption (moderate)^3^ | 8 | 0.8 | 8 | 0.8 | 12.8 | 1.28 | 11.2 | 1.12 | 12.8 | 1.28 | 11.2 | 1.12 | 9.6 | 0.96 | 11.2 | 1.12 |
| Iron (mg/day) 5% absorption (low)^3^ | 16 | 1.6 | 16 | 1.6 | 25.6 | 2.56 | 22.4 | 2.24 | 25.6 | 2.56 | 22.4 | 2.24 | 19.2 | 1.92 | 22.4 | 2.24 |
| Niacin (mg/day)^4^ | 6 | 0.9 | 6 | 0.9 | 9 | 1.35 | 9 | 1.35 | 12 | 1.8 | 11 | 1.65 | 12 | 1.8 | 11 | 1.65 |
| Riboflavin (mg/day)^4^ | 0.8 | 0.08 | 0.8 | 0.08 | 1.1 | 0.11 | 1.1 | 0.11 | 1.4 | 0.14 | 1.4 | 0.14 | 1.3 | 0.13 | 1.3 | 0.13 |
| Thiamin (mg/day)^4^ | 0.5 | 0.05 | 0.5 | 0.05 | 0.7 | 0.07 | 0.7 | 0.07 | 1 | 0.1 | 0.9 | 0.09 | 1 | 0.1 | 0.9 | 0.09 |
| Vitamin A (RE/day)^5^ | 320 | 64 | 320 | 64 | 480 | 96 | 480 | 96 | 580 | 116 | 490 | 98 | 570 | 114 | 490 | 98 |
| Vitamin B6 (mg/day)^3^ | 0.9 | 0.09 | 0.9 | 0.09 | 1.2 | 1.12 | 1.2 | 1.12 | 1.5 | 0.15 | 1.3 | 0.13 | 1.5 | 0.15 | 1.3 | 0.13 |
| Vitamin B12 (µg/day)^3^ | 1 | 0.1 | 1 | 0.1 | 1.5 | 0.15 | 1.5 | 0.15 | 2.0 | 0.2 | 2 | 0.2 | 2.0 | 0.2 | 2.0 | 0.2 |
| Vitamin C (mg/day)^3^ | 40 | 4 | 40 | 4 | 60 | 6 | 60 | 6 | 85 | 8.5 | 75 | 7.5 | 90 | 9 | 80 | 8 |
| Zinc (mg/day) 300 mg/day phytate (refined diet)^2^ | NA | NA | NA | NA | NA | NA | NA | NA | NA | NA | NA | NA | 7.5 | 0.93 | 6.2 | 0.78 |
| Zinc (mg/day) 600 mg/day phytate (semi-refined diet)^2^ | NA | NA | NA | NA | NA | NA | NA | NA | NA | NA | NA | NA | 9.3 | 1.16 | 7.6 | 1.16 |
| Zinc (mg/day) 900 mg/day phytate (semi-unrefined diet)^2^ | 6.2 | 0.78 | 6.2 | 0.78 | 8.9 | 1.11 | 8.9 | 1.11 | 11.8 | 1.48 | 9.9 | 1.24 | 11 | 1.38 | 8.9 | 1.11 |
| Zinc (mg/day) 1,200 mg/day phytate (unrefined diet)^2^ | NA | NA | NA | NA | NA | NA | NA | NA | NA | NA | NA | NA | 12.7 | 1.59 | 10.2 | 1.28 |

^1^The coefficients of variation (CVs) were used from the FAO and WHO, if available (58), or otherwise from the National Academy of Medicine (57).

^2^CV is 12.5%.

^3^CV is 10%.

^4^CV is 15%.

^5^CV is 20%.

| Burkina Faso | Low | Low absorption | Unrefined diet |
| --- | --- | --- | --- |
| Ethiopia | Low | Low absorption | Unrefined diet |
| Kenya | Medium | Moderate absorption | Semi-unrefined diet |
| Mozambique | Low | Low absorption | Unrefined diet |
| Nigeria | Low | Low absorption | Unrefined diet |
| Uganda | Low | Low absorption | Unrefined diet |
| Zambia | Medium | Moderate absorption | Semi-unrefined diet |
| Argentina | High | High absorption | Semi-refined diet |
| Bolivia | High | High absorption | Semi-refined diet |
| Brazil | High | High absorption | Semi-refined diet |
| Mexico | High | High absorption | Semi-refined diet |
| Bangladesh | Medium | Moderate absorption | Semi-unrefined diet |
| India | Medium | Moderate absorption | Semi-unrefined diet |
| Lao PDR | Medium | Moderate absorption | Semi-unrefined diet |
| Philippines | High | High absorption | Semi-refined diet |
| Bulgaria | Very high | High absorption | Refined diet |
| Italy | Very high | High absorption | Refined diet |
| Romania | Very high | High absorption | Refined diet |

**Supplementary table 2. Human development index (HDI), iron absorption, and zinc bioavailability categories for the FAO/WHO GIFT survey countries evaluated^1^**

^1^GIFT, Global Individual Food consumption data Tool; PDR, People’s Democratic Republic

^2^Low, moderate, and high absorption represent 5%, 10%, and 16% absorption, respectively

^3^Refined, semi-refined, semi-unrefined, and unrefined diets include 300, 600, 900, and 1200 mg phytate/day, respectively.

|  | **Mexico ‘12** | **Brazil ‘13** | **Bolivia ‘09** | **Argentina ‘12** | **Italy ’05** | **Romania ‘12 (adults)^4^** | **Romania ‘12 (children)** | **Bulgaria ‘04** | **India ‘09** | **Lao PDR ‘16** | **Total** |
| --- | --- | --- | --- | --- | --- | --- | --- | --- | --- | --- | --- |
| **All starchy staples** | 97.2 | 99.4 | 100 | 100 | 100 | 100 | 99.7 | 98.3 | 100 | 100 | 99.4 |
| **Beans and peas** | 34.1 | 70.2 | 26.3 | 0 | 17.3 | 0 | 13.6 | 23.3 | 62.6 | 1.1 | 67.2 |
| **Nuts and seeds** | 2.2 | 0.9 | 21.1 | 0 | 0.7 | 0 | 3.5 | 10.3 | 2.4 | 1.1 | 1.2 |
| **Dairy** | 63.9 | 64.4 | 26.3 | 90 | 89.9 | 50.0 | 77.4 | 80.2 | 56.7 | 4.2 | 63.5 |
| **Flesh foods** | 81.4 | 95.1 | 84.2 | 80.0 | 98.6 | 100 | 87.1 | 87.1 | 14.6 | 91.6 | 87.1 |
| **Egg** | 41.8 | 20.1 | 31.6 | 40.0 | 28.8 | 50.0 | 50.2 | 32.8 | 4.7 | 28.4 | 19.8 |
| **Dark green leafy vegetables** | 0.7 | 2.4 | 10.5 | 0 | 18.7 | 0 | 1.7 | 0.9 | 8.6 | 40.0 | 3.1 |
| **Vitamin A-rich fruits and vegetables** | 12.6 | 20.7 | 26.3 | 20.0 | 13.7 | 100 | 80.1 | 6.9 | 7.6 | 22.1 | 19.6 |
| **Other vegetables** | 83.5 | 84.0 | 89.5 | 80.0 | 87.1 | 100 | 93.0 | 95.7 | 80.2 | 51.6 | 83.6 |
| **Other fruits** | 44.1 | 19.5 | 42.1 | 10.0 | 66.9 | 0 | 59.6 | 20.7 | 11.1 | 33.7 | 20.2 |
| **FGDS (0-10 points)^2^** | 5 (4, 5)  4.6 ± 1.3 | 5 (4, 6)  4.8 ± 1.2 | 4 (4, 5)  4.6 ± 1.1 | 4 (4, 5)  4.2 ± 1.1 | 5 (4, 6)  5.2 ± 1.2 | 5 (4, 6)  5.0 ± 1.4 | 6 (5, 7)  5.7 ± 1.2 | 4 (4, 5)  4.6 ± 1.2 | 3 (3, 4)  3.5 ± 1.1 | 4 (3, 4)  3.7 ± 1.2 | 5 (4, 5)  4.6 ± 1.3 |
| ***Inter*-person SD** | 1.3 | 1.2 | 1.1 | 1.1 | 1.2 | 1.4 | 1.2 | 1.2 | 1.1 | 1.1 | 1.3 |
| ***Intra*-person SD^3^** | 0.8 (121) | 0.8 (2,179) | 0.9 (19) | **NA** | 0.9 (139) | 0.7 (2) | **NA** | **NA** | **NA** | 0.7 (13) | 0.8 (2,473) |
| **MDD-W (≥5 food groups)** | 54.1 | 60.3 | 42.1 | 40.0 | 71.2 | 50.0 | 83.6 | 49.1 | 14.8 | 24.2 | 56.0 |
| **Sample size** | 1,411 | 32,059 | 19 | 10 | 139 | 2 | 287 | 116 | 3,457 | 95 | 37,595 |

**Supplemental table 3. Food group diversity score (FGDS) and the proportion of adolescent boys (10-19 years) consuming individual food groups and achieving MDD-W, by survey on FAO/ WHO GIFT^1^**

^1^Data are percentages unless otherwise stated. GIFT, Global Individual Food consumption data Tool; MDD-W, Minimum Dietary Diversity for Women; PDR, People’s Democratic Republic; SD, standard deviation.

^2^Median (*P*^25^,*P*^75^) and mean ± SD of the first dietary recall.

^3^Among individuals with ≥2 dietary recalls (*n*), using Stata’s *xtsum* command.

^4^Includes individuals aged 19 years.

**Supplemental table 4. Food group diversity score (FGDS) and the proportion of non-pregnant non-lactating adolescent girls (10-19 years) consuming individual food groups and achieving MDD-W, by survey on FAO/ WHO GIFT^1^**

|  | **Mexico ‘12** | **Brazil ‘13** | **Bolivia ‘09** | **Argentina ‘12** | **Italy ‘05** | **Romania ‘12 (adults)^4^** | **Romania ‘12 (children)** | **Bulgaria ‘04** | **Burkina Faso ‘10** | **Nigeria ‘11** | **Uganda ‘07** | **Tanzania ‘16** |
| --- | --- | --- | --- | --- | --- | --- | --- | --- | --- | --- | --- | --- |
| **All starchy staples** | 96.9 | 98.7 | 100 | 93.6 | 98.8 | 100 | 96.8 | 96.2 | 100 | 100 | 100 | 100 |
| **Beans and peas** | 29.1 | 60.2 | 31.3 | 8.5 | 12.4 | 0 | 8.5 | 9.4 | 100 | 41.7 | 0 | 0 |
| **Nuts and seeds** | 2.1 | 1.0 | 6.3 | 6.4 | 0 | 0 | 3.6 | 8.5 | 0 | 37.5 | 0 | 100 |
| **Dairy** | 67.4 | 59.8 | 43.8 | 66.0 | 92.0 | 100 | 77.2 | 75.5 | 100 | 12.5 | 0 | 100 |
| **Flesh foods** | 75.9 | 93.5 | 93.8 | 85.1 | 90.7 | 50.0 | 80.1 | 79.3 | 0 | 95.8 | 0 | 100 |
| **Egg** | 35.6 | 19.4 | 37.5 | 34.0 | 31.5 | 50.0 | 48.8 | 25.5 | 0 | 0 | 0 | 0 |
| **Dark green leafy vegetables** | 1.1 | 2.7 | 0 | 8.5 | 20.4 | 0 | 2.1 | 2.8 | 100 | 50.0 | 100 | 0 |
| **Vitamin A-rich fruits and vegetables** | 14.2 | 21.7 | 25.0 | 10.6 | 11.7 | 100 | 73.7 | 7.6 | 0 | 12.5 | 0 | 0 |
| **Other vegetables** | 82.5 | 75.8 | 93.8 | 68.1 | 88.3 | 100 | 89.7 | 90.6 | 100 | 91.7 | 100 | 100 |
| **Other fruits** | 46.0 | 22.0 | 37.5 | 27.7 | 77.2 | 100 | 61.9 | 22.6 | 0 | 58.3 | 100 | 0 |
| **FGDS (0-10 points)^2^** | 5 (4, 5)  4.5 ± 1.3 | 5 (4, 5)  4.6 ± 1.3 | 5 (4, 6)  4.7 ± 1.0 | 4 (3, 5)  4.1 ± 1.4 | 5 (4, 6)  5.2 ± 1.1 | 6 (5, 7)  6.0 ± 1.4 | 6 (5, 6)  5.4 ± 1.4 | 4 (4, 5)  4.2 ± 1.1 | 5 (5, 5)  5.0 | 5 (4, 5)  5.0 ± 1.2 | 4 (4, 4)  4.0 | 4 (4, 4)  4.0 |
| ***Inter*-person SD** | 1.3 | 1.3 | 1.0 | 1.4 | 1.1 | 1.4 | 1.4 | 1.1 | **NA** | 1.2 | **NA** | **NA** |
| ***Intra*-person SD^3^** | 0.8 (123) | 0.8 (2,807) | 0.7 (16) | **NA** | 0.7 (162) | 1.3 (2) | **NA** | **NA** | **NA** | 0.9 (4) | **NA** | **NA** |
| **MDD-W (≥5 food groups)** | 51.6 | 52.4 | 56.3 | 36.2 | 74.7 | 100 | 76.5 | 36.8 | 100 | 70.8 | 0 | 0 |
| **Sample size** | 1,369 | 39,912 | 16 | 47 | 162 | 2 | 281 | 106 | 1 | 24 | 1 | 1 |

^1^Data are percentages unless otherwise stated. GIFT, Global Individual Food consumption data Tool; MDD-W, Minimum Dietary Diversity for Women; SD, standard deviation.

^2^Median (*P*^25^,*P*^75^) and mean ± SD of the first dietary recall.

^3^Among individuals with ≥2 dietary recalls (*n*), using Stata’s *xtsum* command.

^4^Includes individuals aged 19 years.

**Supplemental table 4 cont’d. Food group diversity score (FGDS) and the proportion of non-pregnant non-lactating adolescent girls (10-19 years) consuming individual food groups and achieving MDD-W, by survey on FAO/ WHO GIFT^1^**

|  | **Zambia ‘09** | **Mozambique ‘10** | **Ethiopia ‘13 (South)** | **Kenya ‘12** | **Kenya ‘14**  **(lean)** | **Kenya ‘15 (baseline)** | **Kenya ‘15**  **(plenty)** | **Kenya ‘16** | **Kenya ‘18** | **India ‘09** | **Bangladesh ‘07** | **Lao PDR ‘16** | **Total** |
| --- | --- | --- | --- | --- | --- | --- | --- | --- | --- | --- | --- | --- | --- |
| **All starchy staples** | 100 | 99.3 | 100 | 85.7 | 100 | 80.0 | 100 | 94.4 | 92.9 | 100 | 100 | 100 | 99.0 |
| **Beans and peas** | 25.0 | 24.3 | 75.0 | 14.3 | 0 | 20.0 | 0 | 66.7 | 35.7 | 61.0 | 20.0 | 1.0 | 58.2 |
| **Nuts and seeds** | 25.0 | 10.1 | 0 | 28.6 | 0 | 0 | 0 | 0 | 0 | 2.9 | 0 | 0 | 1.3 |
| **Dairy** | 0 | 1.4 | 25.0 | 42.9 | 83.3 | 80.0 | 50.0 | 72.2 | 50.0 | 54.3 | 20.0 | 7.1 | 59.2 |
| **Flesh foods** | 75.0 | 68.0 | 0 | 28.6 | 66.7 | 40.0 | 50.0 | 22.2 | 28.6 | 13.8 | 40.0 | 95.9 | 86.0 |
| **Egg** | 0 | 7.7 | 0 | 0 | 0 | 0 | 0 | 0 | 0 | 4.0 | 20.0 | 14.3 | 18.7 |
| **Dark green leafy vegetables** | 50.0 | 28.5 | 25.0 | 57.1 | 100 | 60.0 | 50.0 | 44.4 | 64.3 | 7.6 | 60.0 | 48.0 | 3.6 |
| **Vitamin A-rich fruits and vegetables** | 75.0 | 44.6 | 0 | 0 | 0 | 0 | 0 | 0 | 21.4 | 7.6 | 20.0 | 25.5 | 20.8 |
| **Other vegetables** | 100 | 64.3 | 100 | 71.4 | 100 | 60.0 | 100 | 55.6 | 64.3 | 79.3 | 100 | 55.1 | 76.3 |
| **Other fruits** | 0 | 33.4 | 0 | 57.1 | 16.7 | 20.0 | 0 | 0 | 0 | 9.8 | 0 | 39.8 | 22.3 |
| **FGDS (0-10 points)^2^** | 4 (3, 6)  4.5 ± 1.9 | 4 (3, 5)  3.8 ± 1.1 | 3 (3, 4)  3.3 ± 1.3 | 4 (2, 5)  3.9 ± 1.3 | 5 (4, 5)  4.7 ± 0.5 | 4 (3, 4)  3.6 ± 1.1 | 4 (3, 4)  3.5 ± 0.7 | 4 (3, 4)  3.6 ± 1.2 | 4 (3, 5)  3.6 ± 1.4 | 4 (3, 4)  3.4 ± 1.0 | 3 (3, 5)  3.8 ± 1.6 | 4 (3, 4)  3.9 ± 1.1 | 4 (4, 5)  4.5 ± 1.3 |
| ***Inter*-person SD** | 1.9 | 1.1 | 1.3 | 1.3 | 0.5 | 1.1 | 0.7 | 1.2 | 1.4 | 1.0 | 1.6 | 1.1 | 1.3 |
| ***Intra*-person SD^3^** | 0.4 (3) | 0.9 (87) | 0.4 (2) | **NA** | 0.7 (4) | 1.2 (4) | 0 (2) | 0.6 (16) | 0.8 (8) | **NA** | 0.7 (5) | 0.5 (9) | 0.8 (3,255) |
| **MDD-W (≥5 food groups)** | 50.0 | 26.6 | 25.0 | 42.9 | 66.7 | 20 | 0 | 22.2 | 28.6 | 12.5 | 40 | 23.5 | 49.1 |
| **Sample size** | 4 | 428 | 4 | 7 | 6 | 5 | 2 | 18 | 14 | 3,827 | 5 | 98 | 46,340 |

^1^Data are percentages unless otherwise stated. GIFT, Global Individual Food consumption data Tool; MDD-W, Minimum Dietary Diversity for Women; PDR, People’s Democratic Republic; SD, standard deviation.

^2^Median (*P*^25^,*P*^75^) and mean ± SD of the first dietary recall.

^3^Among individuals with ≥2 dietary recalls (*n*), using Stata’s *xtsum* command.

|  | **Mexico ‘12** | **Brazil ‘13** | **Bolivia ‘09** | **Argentina ‘12** | **Italy ’05** | **Romania ‘12 (adults)^2^** | **Romania ‘12 (children)** | **Bulgaria ‘04** | **India ‘09** | **Lao PDR ‘16** | **Total** |
| --- | --- | --- | --- | --- | --- | --- | --- | --- | --- | --- | --- |
| **Energy (kcal/d)** | 2116 ± 882 | 1803 ± 971 | 1510 ± 421 | 2355 ± 877 | 2610 ± 875 | 2190 ± 486 | 2304 ± 1007 | 2742 ± 1012 | 1571 ± 565 | 1758 ± 727 | 1803 ± 945 |
| **Calcium (NAR)** | 0.82 ± 0.22 | 0.42 ± 0.27 | 0.42 ± 0.17 | 0.63 ± 0.23 | 0.79 ± 0.23 | 0.70 ± 0.42 | 0.87 ± 0.23 | 0.63 ± 0.28 | 0.28 ± 0.21 | 0.25 ± 0.24 | 0.43 ± 0.28 |
| **Iron (NAR)** | 0.98 ± 0.09 | 0.91 ± 0.18 | 0.97 ± 0.10 | 1.00 ± 0.00 | 0.98 ± 0.08 | 1.00 ± 0.00 | 0.97 ± 0.09 | 0.98 ± 0.08 | 0.76 ± 0.25 | 0.56 ± 0.22 | 0.90 ± 0.19 |
| **Zinc (NAR)** | 0.84 ± 0.21 | 0.78 ± 0.25 | 0.72 ± 0.15 | 0.88 ± 0.21 | **NA** | 1.00 ± 0.00 | 0.79 ± 0.24 | 0.84 ± 0.19 | 0.69 ± 0.24 | **NA** | 0.78 ± 0.25 |
| **Vitamin A (NAR)** | 0.65 ± 0.33 | 0.36 ± 0.31 | 0.40 ± 0.25 | 0.75 ± 0.30 | 0.85 ± 0.22 | 1.00 ± 00 | 0.92 ± 0.20 | 0.88 ± 0.21 | 0.21 ± 0.28 | 0.30 ± 0.30 | 0.36 ± 0.32 |
| **Thiamin (NAR)** | 0.93 ± 0.16 | 0.79 ± 0.27 | 0.92 ± 0.17 | 1.00 ± 0.00 | 0.94 ± 0.14 | 1.00 ± 0.00 | 0.96 ± 0.12 | 0.90 ± 0.17 | 0.86 ± 0.20 | 0.56 ± 0.29 | 0.80 ± 0.26 |
| **Riboflavin (NAR)** | 0.84 ± 0.23 | 0.70 ± 0.33 | 0.68 ± 0.24 | 0.91 ± 0.17 | 0.95 ± 0.13 | 0.93 ± 0.10 | 0.92 ± 0.17 | 0.81 ± 0.25 | 0.51 ± 0.22 | 0.46 ± 0.30 | 0.69 ± 0.32 |
| **Niacin (NAR)** | 0.87 ± 0.21 | 0.80 ± 0.27 | 0.97 ± 0.09 | 0.94 ± 0.13 | 1.00 ± 0.02 | 1.00 ± 0.00 | 0.96 ± 0.12 | 0.99 ± 0.04 | 0.78 ± 0.22 | 0.82 ± 0.23 | 0.80 ± 0.26 |
| **Vitamin B6 (NAR)** | 0.74 ± 0.28 | 0.50 ± 0.33 | 0.90 ± 0.12 | 0.64 ± 0.31 | 0.99 ± 0.05 | 1.00 ± 0.00 | 0.91 ± 0.17 | 0.92 ± 0.15 | **NA** | **NA** | 0.52 ± 0.34 |
| **Folate (NAR)** | 0.85 ± 0.25 | 0.91 ± 0.20 | 0.84 ± 0.23 | 1.00 ± 0.00 | 0.95 ± 0.13 | 0.95 ± 0.07 | 0.94 ± 0.14 | 0.80 ± 0.24 | 0.63 ± 0.25 | **NA** | 0.89 ± 0.22 |
| **Vitamin B12 (NAR)** | 0.81 ± 0.30 | 0.85 ± 0.27 | 0.72 ± 0.35 | 0.89 ± 0.22 | 0.98 ± 0.09 | 1.00 ± 0.00 | 0.87 ± 0.25 | 0.81 ± 0.30 | **NA** | **NA** | 0.77 ± 0.35 |
| **Vitamin C (NAR)** | 0.66 ± 0.37 | 0.53 ± 0.43 | 0.62 ± 0.29 | 0.48 ± 0.37 | 0.82 ± 0.28 | 0.98 ± 0.03 | 0.83 ± 0.27 | 0.81 ± 0.27 | 0.43 ± 0.32 | 0.43 ± 0.36 | 0.53 ± 0.42 |
| **MAR** | 0.82 ± 0.17 | 0.69 ± 0.18 | 0.74 ± 0.11 | 0.83 ± 0.13 | 0.92 ± 0.09 | 0.96 ± 0.05 | 0.90 ± 0.12 | 0.85 ± 0.14 | 0.57 ± 0.17 | 0.48 ± 0.19 | 0.68 ± 0.19 |
| **MAR >0.60** | 1,235 (87.5) | 22,518 (70.2) | 17 (89.5) | 9 (90.0) | 138 (99.3) | 2 (100) | 275 (95.8) | 109 (94.0) | 1,603 (46.4) | 22 (23.2) | 25,928 (69.0) |
| **Sample size** | 1,411 | 32,059 | 19 | 10 | 139 | 2 | 287 | 116 | 3,457 | 95 | 37,595 |

**Supplemental table 5. Energy intakes, NARs of 11 micronutrients, and MAR among adolescent boys (10-19 years), by survey on FAO/ WHO GIFT^1^**

^1^Data are mean ± SD or frequency (%) from the first quantitative 24-h recall. GIFT, Global Individual Food consumption data Tool; MAR, Mean Adequacy Ratio; NAR, Nutrient Adequacy Ratio; PDR, People’s Democratic Republic.

^2^Includes individuals aged 19 years.

**Supplemental table 6. Accuracy of food group cut-offs for an MAR >0.6 among adolescent boys (10-19 years), by survey on FAO/ WHO GIFT^1^**

| **Country** | **Mexico ‘12 (*n*=1,411)** | | | | | **Brazil ‘13 (*n*=32,059)** | | | | | **Bolivia ‘09 (*n*=19)** | | | | |
| --- | --- | --- | --- | --- | --- | --- | --- | --- | --- | --- | --- | --- | --- | --- | --- |
| **Food group cut-off** | Sensitivity | Specificity | PCC | LR+ | LR- | Sensitivity | Specificity | PCC | LR+ | LR- | Sensitivity | Specificity | PCC | LR+ | LR- |
| **≥ 1** | 100 | 0.00 | 87.5 | 1.00 | NA | 99.9 | 0.38 | 70.3 | 1.00 | 0.15 | NA | NA | NA | NA | NA |
| **≥ 2** | 99.8 | 6.25 | 88.1 | 1.06 | 0.04 | 99.8 | 1.57 | 70.5 | 1.01 | 0.16 | NA | NA | NA | NA | NA |
| **≥ 3** | 97.8 | 18.2 | 87.9 | 1.20 | 0.12 | 98.0 | 7.25 | 71.0 | 1.06 | 0.27 | 100 | 0.00 | 89.5 | 1.00 | NA |
| **≥ 4** | 86.6 | 53.4 | 82.5 | 1.86 | 0.25 | 90.9 | 22.7 | 70.6 | 1.18 | 0.40 | 88.2 | 0.00 | 79.0 | 0.88 | NA |
| **≥ 5** | 59.4 | 83.0 | 62.3 | 3.48 | 0.49 | 67.0 | 55.6 | 63.6 | 1.51 | 0.59 | 47.1 | 100 | 52.6 | NA | 0.53 |
| **≥ 6** | 27.0 | 96.6 | 35.7 | 7.93 | 0.76 | 30.1 | 85.3 | 46.5 | 2.04 | 0.82 | 23.5 | 100 | 31.6 | NA | 0.76 |
| **≥ 7** | 6.72 | 100 | 18.4 | NA | 0.93 | 8.51 | 97.2 | 34.9 | 3.02 | 0.94 | 5.88 | 100 | 15.8 | NA | 0.94 |
| **≥ 8** | 0.97 | 100 | 13.3 | NA | 0.99 | 1.44 | 99.8 | 30.7 | 7.25 | 0.99 | 0.00 | 100 | 10.5 | NA | 1.00 |
| **≥ 9** | 0.08 | 100 | 12.5 | NA | 1.00 | 0.12 | 100 | 29.8 | 11.4 | 1.00 | NA | NA | NA | NA | NA |
| **>9** | 0.00 | 100 | 12.5 | NA | 1.00 | 0.00 | 100 | 29.8 | NA | 1.00 | NA | NA | NA | NA | NA |
| **AUC** | 0.79 (95% CI: 0.75, 0.82) | | | | | 0.65 (95% CI: 0.64, 0.65) | | | | | 0.69 (95% CI: 0.51, 0.84) | | | | |

^1^Values are percentages (except for the AUC values). AUC, area under the curve; LR+; positive likelihood ratio; LR-, negative likelihood ratio; MAR, Mean Adequacy Ratio; NA, not applicable; PCC, percentage correctly classified.

**Supplemental table 6 cont’d. Accuracy of food group cut-offs for an MAR >0.6 among adolescent boys (10-19 years), by survey on FAO/ WHO GIFT^1^**

| **Country** | **Argentina ‘12 (*n*=10)** | | | | | **Italy ‘05 (*n*=139)** | | | | | **Romania ‘12 (*n*=287)** | | | | |
| --- | --- | --- | --- | --- | --- | --- | --- | --- | --- | --- | --- | --- | --- | --- | --- |
| **Food group cut-off** | Sensitivity | Specificity | PCC | LR+ | LR- | Sensitivity | Specificity | PCC | LR+ | LR- | Sensitivity | Specificity | PCC | LR+ | LR- |
| **≥ 1** | NA | NA | NA | NA | NA | NA | NA | NA | NA | NA | NA | NA | NA | NA | NA |
| **≥ 2** | 100 | 0.00 | 90.0 | 1.00 | NA | 100 | 0.00 | 99.3 | 1.00 | NA | 100 | 0.00 | 95.8 | 1.00 | NA |
| **≥ 3** | 88.9 | 0.00 | 80.0 | 0.89 | NA | 100 | 100 | 100 | NA | 0.00 | 99.6 | 8.33 | 95.8 | 1.09 | 0.04 |
| **≥ 4** | 77.8 | 0.00 | 70.0 | 0.78 | NA | 94.9 | 100 | 95.0 | NA | 0.05 | 94.6 | 33.3 | 92.0 | 1.42 | 0.16 |
| **≥ 5** | 44.4 | 100 | 50.0 | NA | 0.56 | 71.7 | 100 | 71.9 | NA | 0.28 | 85.5 | 58.3 | 84.3 | 2.05 | 0.25 |
| **≥ 6** | 11.1 | 100 | 20.0 | NA | 0.89 | 13.8 | 100 | 14.4 | NA | 0.86 | 63.3 | 91.7 | 64.5 | 7.59 | 0.40 |
| **≥ 7** | 0.00 | 100 | 10.0 | NA | 1.00 | 1.45 | 100 | 2.16 | NA | 0.99 | 26.2 | 100 | 29.3 | NA | 0.74 |
| **≥ 8** | NA | NA | NA | NA | NA | 0.00 | 100 | 0.72 | NA | 1.00 | 3.64 | 100 | 7.67 | NA | 0.96 |
| **≥ 9** | NA | NA | NA | NA | NA | 100 | 0.00 | 99.3 | 1.00 | NA | 0.00 | 100 | 4.18 | NA | 1.00 |
| **>9** | NA | NA | NA | NA | NA | NA | NA | NA | NA | NA | NA | NA | NA | NA | NA |
| **AUC** | 0.61 (95% CI: NA) | | | | | 1.00 (95% CI: NA) | | | | | 0.83 (95% CI: 0.74, 0.93) | | | | |

^1^Values are percentages (except for the AUC values). AUC, area under the curve; LR+; positive likelihood ratio; LR-, negative likelihood ratio; MAR, Mean Adequacy Ratio; NA, not applicable; PCC, percentage correctly classified.

**Supplemental table 6 cont’d. Accuracy of food group cut-offs for an MAR >0.6 among adolescent boys (10-19 years), by survey on FAO/ WHO GIFT^1^**

| **Country** | **Bulgaria ‘04 (*n*=116)** | | | | | **India ‘09 (*n*=3,457)** | | | | | **Lao PDR ‘16 (*n*=95)** | | | | |
| --- | --- | --- | --- | --- | --- | --- | --- | --- | --- | --- | --- | --- | --- | --- | --- |
| **Food group cut-off** | Sensitivity | Specificity | PCC | LR+ | LR- | Sensitivity | Specificity | PCC | LR+ | LR- | Sensitivity | Specificity | PCC | LR+ | LR- |
| **≥ 1** | NA | NA | NA | NA | NA | 100 | 0.00 | 46.4 | 1.00 | NA | NA | NA | NA | NA | NA |
| **≥ 2** | 100 | 0.00 | 94.0 | 1.00 | NA | 99.3 | 2.70 | 47.5 | 1.02 | 0.28 | 100 | 0.00 | 23.2 | 1.00 | NA |
| **≥ 3** | 98.2 | 28.6 | 94.0 | 1.37 | 0.06 | 90.5 | 21.5 | 53.5 | 1.15 | 0.44 | 90.9 | 20.6 | 36.8 | 1.14 | 0.44 |
| **≥ 4** | 88.1 | 71.4 | 87.1 | 3.08 | 0.17 | 58.1 | 61.7 | 60.0 | 1.52 | 0.68 | 77.3 | 49.3 | 55.8 | 1.52 | 0.46 |
| **≥ 5** | 52.3 | 100 | 55.2 | NA | 0.48 | 20.3 | 90.0 | 57.7 | 2.04 | 0.88 | 54.6 | 84.9 | 77.9 | 3.62 | 0.53 |
| **≥ 6** | 22.0 | 100 | 26.7 | NA | 0.78 | 5.43 | 98.4 | 55.3 | 3.35 | 0.96 | 22.7 | 95.9 | 79.0 | 5.53 | 0.81 |
| **≥ 7** | 5.50 | 100 | 11.2 | NA | 0.95 | 1.00 | 100 | 54.1 | 18.5 | 0.99 | 9.09 | 100 | 79.0 | NA | 0.91 |
| **≥ 8** | 0.00 | 100 | 6.03 | NA | 1.00 | 0.12 | 100 | 53.7 | NA | 1.00 | 0.00 | 100 | 76.8 | NA | 1.00 |
| **≥ 9** | NA | NA | NA | NA | NA | 0.00 | 100 | 53.6 | NA | 1.00 | NA | NA | NA | NA | NA |
| **>9** | NA | NA | NA | NA | NA | NA | NA | NA | NA | NA | NA | NA | NA | NA | NA |
| **AUC** | 0.88 (95% CI: 0.78, 0.98) | | | | | 0.63 (95% CI: 0.61, 0.64) | | | | | 0.72 (95% CI: 0.59, 0.85) | | | | |

^1^Values are percentages (except for the AUC values). AUC, area under the curve; LR+; positive likelihood ratio; LR-, negative likelihood ratio; MAR, Mean Adequacy Ratio; NA, not applicable; PCC, percentage correctly classified; PDR, People’s Democratic Republic.

|  | **Mexico ‘12** | **Brazil ‘13** | **Bolivia ‘09** | **Argentina ‘12** | **Italy ‘05** | **Romania ‘12 (adults)^2^** | **Romania ‘12 (children)** | **Bulgaria ‘04** | **Burkina Faso ‘10** | **Nigeria ‘11** | **Uganda ‘07** | **Tanzania ‘16** |
| --- | --- | --- | --- | --- | --- | --- | --- | --- | --- | --- | --- | --- |
| **Energy (kcal/d)** | 1787 ± 729 | 1540 ± 913 | 1438 ± 453 | 1818 ± 612 | 2145 ± 642 | 2448 ± 46 | 2031 ± 907 | 1883 ± 929 | 4523 | 2226 ± 797 | 1232 | 2460 |
| **Calcium (NAR)** | 0.77 ± 0.25 | 0.38 ± 0.27 | 0.43 ± 0.22 | 0.52 ± 0.32 | 0.79 ± 0.23 | 0.77 ± 0.33 | 0.80 ± 0.27 | 0.51 ± 0.30 | 1.00 | 0.41 ± 0.13 | 0.40 | 0.77 |
| **Iron (NAR)** | 0.96 ± 0.12 | 0.87 ± 0.21 | 0.98 ± 0.08 | 0.96 ± 0.12 | 0.97 ± 0.09 | 1.00 ± 0.00 | 0.95 ± 0.16 | 0.83 ± 0.22 | 1.00 | 0.59 ± 0.20 | 0.32 | 1.00 |
| **Zinc (NAR)** | 0.79 ± 0.24 | 0.72 ± 0.27 | 0.80 ± 0.18 | 0.90 ± 0.19 | **NA** | 0.83 ± 0.23 | 0.76 ± 0.25 | 0.70 ± 0.27 | 1.00 | 0.91 ± 0.11 | 0.41 | 1.00 |
| **Vitamin A (NAR)** | 0.65 ± 0.33 | 0.36 ± 0.31 | 0.53 ± 0.31 | 0.63 ± 0.35 | 0.91 ± 0.18 | 1.00 ± 0.00 | 0.88 ± 0.24 | 0.86 ± 0.24 | **NA** | 0.00 ± 0.00 | 1.00 | **NA** |
| **Thiamin (NAR)** | 0.89 ± 0.19 | 0.74 ± 0.29 | 0.93 ± 0.10 | 0.91 ± 0.18 | 0.93 ± 0.13 | 1.00 ± 0.00 | 0.94 ± 0.17 | 0.76 ± 0.26 | 1.00 | 1.00 ± 0.00 | 0.51 | 1.00 |
| **Riboflavin (NAR)** | 0.79 ± 0.26 | 0.63 ± 0.34 | 0.71 ± 0.21 | 0.82 ± 0.19 | 0.94 ± 0.13 | 0.75 ± 0.07 | 0.88 ± 0.21 | 0.66 ± 0.26 | 1.00 | 0.71 ± 0.18 | 0.85 | 0.92 |
| **Niacin (NAR)** | 0.83 ± 0.23 | 0.75 ± 0.29 | 0.96 ± 0.08 | 0.91 ± 0.20 | 0.97 ± 0.10 | 0.89 ± 0.15 | 0.94 ± 0.17 | 0.95 ± 0.15 | 1.00 | 0.96 ± 0.11 | 0.49 | **NA** |
| **Vitamin B6 (NAR)** | 0.70 ± 0.29 | 0.47 ± 0.33 | 0.93 ± 0.12 | 0.69 ± 0.34 | 0.97 ± 0.08 | 0.79 ± 0.30 | 0.87 ± 0.22 | 0.83 ± 0.23 | 1.00 | 0.85 ± 0.18 | 0.89 | 1.00 |
| **Folate (NAR)** | 0.80 ± 0.27 | 0.85 ± 0.25 | 0.81 ± 0.18 | 0.91 ± 0.21 | 0.94 ± 0.13 | 0.82 ± 0.26 | 0.90 ± 0.20 | 0.61 ± 0.27 | 0.72 | 0.86 ± 0.20 | 0.66 | 1.00 |
| **Vitamin B12 (NAR)** | 0.75 ± 0.32 | 0.80 ± 0.30 | 0.82 ± 0.23 | 0.82 ± 0.31 | 0.98 ± 0.10 | 0.54 ± 0.10 | 0.80 ± 0.30 | 0.72 ± 0.32 | 1.00 | 0.95 ± 0.14 | 0.00 | **NA** |
| **Vitamin C (NAR)** | 0.67 ± 0.37 | 0.54 ± 0.42 | 0.70 ± 0.28 | 0.38 ± 0.33 | 0.85 ± 0.24 | 0.95 ± 0.08 | 0.81 ± 0.29 | 0.80 ± 0.29 | 0.41 | 0.91 ± 0.19 | 1.00 | 0.07 |
| **MAR** | 0.78 ± 0.19 | 0.65 ± 0.20 | 0.78 ± 0.08 | 0.77 ± 0.16 | 0.92 ± 0.09 | 0.85 ± 0.00 | 0.87 ± 0.17 | 0.75 ± 0.18 | 0.91 | 0.83 ± 0.10 | 0.59 | 0.85 |
| **MAR >0.60** | 1,114 (81.4) | 24,682 (61.8) | 16 (100) | 41 (87.2) | 159 (98.2) | 2 (100) | 261 (92.9) | 86 (81.1) | 1 (100) | 22 (91.7) | 0 (0.00) | 1 (100) |
| **Sample size** | 1,369 | 39,912 | 16 | 47 | 162 | 2 | 281 | 106 | 1 | 24 | 1 | 1 |

**Supplemental table 7. Energy intakes, NARs of 11 micronutrients, and MAR among non-pregnant non-lactating adolescent girls (10-19 years), by survey on FAO/ WHO GIFT^1^**

^1^Data are mean ± SD or frequency (%) from the first quantitative 24-h recall. GIFT, Global Individual Food consumption data Tool; MAR, Mean Adequacy Ratio; NAR, Nutrient Adequacy Ratio.

^2^Includes individuals aged 19 years.

|  | **Zambia ‘09** | **Mozambique ‘10** | **Ethiopia ‘13 (South)** | **Kenya ‘12** | **Kenya ‘14**  **(lean)** | **Kenya ‘15 (baseline)** | **Kenya ‘15**  **(plenty)** | **Kenya ‘16** | **Kenya ‘18** | **India ‘09** | **Bangladesh ‘07** | **Lao PDR ‘16** | **Total** |
| --- | --- | --- | --- | --- | --- | --- | --- | --- | --- | --- | --- | --- | --- |
| **Energy (kcal/d)** | 1731 ± 338 | 1437 ± 686 | 2234 ± 669 | 1627 ± 607 | 2481 ± 825 | 3156 ± 1698 | 3028 ± 31 | 2635 ± 1619 | 1405 ± 824 | 1478 ± 526 | 1758 ± 485 | 1447 ± 533 | 1549 ± 883 |
| **Calcium (NAR)** | 0.36 ± 0.19 | 0.40 ± 0.26 | 0.73 ± 0.34 | 0.47 ± 0.19 | 0.71 ± 0.30 | 0.44 ± 0.35 | 0.70 ± 0.07 | 0.41 ± 0.24 | 0.63 ± 0.35 | 0.26 ± 0.19 | 0.28 ± 0.20 | 0.25 ± 0.25 | 0.39 ± 0.28 |
| **Iron (NAR)** | 0.90 ± 0.13 | 0.53 ± 0.25 | 1.00 ± 0.00 | 0.90 ± 0.27 | 0.88 ± 0.18 | 1.00 ± 0.00 | 1.00 ± 0.00 | 0.80 ± 0.29 | 0.95 ± 0.18 | 0.77 ± 0.25 | 0.85 ± 0.18 | 0.57 ± 0.27 | 0.86 ± 0.22 |
| **Zinc (NAR)** | 0.80 ± 0.31 | 0.52 ± 0.24 | 0.94 ± 0.12 | 0.80 ± 0.27 | 0.89 ± 0.18 | 0.94 ± 0.14 | 1.00 ± 0.00 | 0.80 ± 0.27 | 0.69 ± 0.30 | 0.69 ± 0.24 | 0.57 ± 0.13 | **NA** | 0.72 ± 0.27 |
| **Vitamin A (NAR)** | 0.84 ± 0.31 | 0.45 ± 0.40 | 0.10 ± 0.11 | 0.64 ± 0.46 | **NA** | **NA** | **NA** | 0.55 ± 0.43 | 0.36 ± 0.34 | 0.21 ± 0.27 | 0.44 ± 0.20 | 0.31 ± 0.30 | 0.36 ± 0.32 |
| **Thiamin (NAR)** | 0.93 ± 0.15 | 0.83 ± 0.22 | 0.87 ± 0.26 | 0.91 ± 0.24 | 0.95 ± 0.12 | 0.89 ± 0.25 | 1.00 ± 0.00 | 0.84 ± 0.22 | 0.68 ± 0.28 | 0.85 ± 0.21 | 0.88 ± 0.21 | 0.59 ± 0.32 | 0.76 ± 0.28 |
| **Riboflavin (NAR)** | 0.67 ± 0.22 | 0.37 ± 0.22 | 1.00 ± 0.00 | 0.74 ± 0.32 | 0.91 ± 0.21 | 0.88 ± 0.27 | 0.80 ± 0.28 | 0.74 ± 0.26 | 0.57 ± 0.29 | 0.47 ± 0.21 | 0.35 ± 0.16 | 0.46 ± 0.33 | 0.62 ± 0.33 |
| **Niacin (NAR)** | 0.97 ± 0.06 | 0.65 ± 0.28 | 0.67 ± 0.33 | 0.98 ± 0.04 | 0.92 ± 0.18 | 0.84 ± 0.35 | 1.00 ± 0.00 | 0.70 ± 0.31 | 0.64 ± 0.27 | 0.76 ± 0.23 | 1.00 ± 0.00 | 0.78 ± 0.25 | 0.76 ± 0.28 |
| **Vitamin B6 (NAR)** | 0.80 ± 0.29 | 0.66 ± 0.28 | **NA** | 0.89 ± 0.30 | 0.93 ± 0.18 | 0.86 ± 0.31 | 0.93 ± 0.10 | 0.75 ± 0.28 | **NA** | **NA** | 1.00 ± 0.00 | **NA** | 0.48 ± 0.33 |
| **Folate (NAR)** | 0.76 ± 0.29 | 0.72 ± 0.30 | 0.87 ± 0.26 | 0.05 ± 0.07 | 0.92 ± 0.16 | 0.93 ± 0.07 | 0.64 ± 0.37 | 0.83 ± 0.24 | 0.71 ± 0.30 | 0.60 ± 0.26 | 0.37 ± 0.12 | **NA** | 0.82 ± 0.26 |
| **Vitamin B12 (NAR)** | 0.37 ± 0.48 | 0.56 ± 0.39 | **NA** | 0.10 ± 0.13 | 0.78 ± 0.27 | 0.51 ± 0.45 | 1.00 ± 0.00 | 0.46 ± 0.37 | 0.35 ± 0.40 | **NA** | 0.30 ± 0.43 | **NA** | 0.73 ± 0.36 |
| **Vitamin C (NAR)** | 0.38 ± 0.25 | 0.81 ± 0.32 | 0.72 ± 0.33 | 0.88 ± 0.21 | 0.99 ± 0.03 | 0.86 ± 0.20 | 0.58 ± 0.58 | 0.82 ± 0.21 | 0.60 ± 0.39 | 0.43 ± 0.32 | 0.80 ± 0.19 | 0.50 ± 0.34 | 0.54 ± 0.41 |
| **MAR** | 0.71 ± 0.14 | 0.59 ± 0.19 | 0.77 ± 0.13 | 0.67 ± 0.17 | 0.89 ± 0.17 | 0.82 ± 0.17 | 0.86 ± 0.13 | 0.70 ± 0.20 | 0.62 ± 0.21 | 0.56 ± 0.17 | 0.62 ± 0.11 | 0.49 ± 0.22 | 0.64 ± 0.20 |
| **MAR >0.60** | 3 (75.0) | 214 (50.0) | 3 (75.0) | 6 (85.7) | 5 (83.3) | 4 (80.0) | 2 (100) | 14 (77.8) | 8 (57.1) | 1,692 (44.2) | 4 (80.0) | 32 (32.7) | 28,373 (61.2) |
| **Sample size** | 4 | 428 | 4 | 7 | 6 | 5 | 2 | 18 | 14 | 3,827 | 5 | 98 | 46,340 |

**Supplemental table 7 cont’d. Energy intakes, NARs of 11 micronutrients, and MAR among non-pregnant non-lactating adolescent girls (10-19 years), by survey on FAO/ WHO GIFT^1^**

^1^Data are mean ± SD or frequency (%) from the first quantitative 24-h recall. GIFT, Global Individual Food consumption data Tool; MAR, Mean Adequacy Ratio; NAR, Nutrient Adequacy Ratio; PDR, People’s Democratic Republic.

**Supplemental table 8. Accuracy of food group cut-offs for an MAR >0.6 among non-pregnant non-lactating adolescent girls (10-19 years), by survey on FAO/ WHO GIFT^1^**

| **Country** | **Mexico ‘12 (*n*=1369)** | | | | | **Brazil ‘13 (*n*=39,912)** | | | | | **Argentina ‘12 (*n*=47)** | | | | |
| --- | --- | --- | --- | --- | --- | --- | --- | --- | --- | --- | --- | --- | --- | --- | --- |
| **Food group cut-off** | Sensitivity | Specificity | PCC | LR+ | LR- | Sensitivity | Specificity | PCC | LR+ | LR- | Sensitivity | Specificity | PCC | LR+ | LR- |
| **≥ 1** | 99.9 | 0.39 | 81.4 | 1.00 | 0.22 | 99.9 | 0.58 | 62.0 | 1.00 | 0.15 | NA | NA | NA | NA | NA |
| **≥ 2** | 99.7 | 5.88 | 82.3 | 1.06 | 0.05 | 99.5 | 2.44 | 62.6 | 1.02 | 0.21 | 100 | 0.00 | 87.2 | 1.00 | NA |
| **≥ 3** | 97.7 | 17.7 | 82.7 | 1.19 | 0.13 | 96.6 | 10.1 | 63.6 | 1.07 | 0.33 | 92.7 | 50.0 | 87.2 | 1.85 | 0.15 |
| **≥ 4** | 86.5 | 51.4 | 79.9 | 1.78 | 0.26 | 86.3 | 29.2 | 64.5 | 1.22 | 0.47 | 70.7 | 100 | 74.5 | NA | 0.29 |
| **≥ 5** | 60.2 | 85.9 | 65.0 | 4.26 | 0.46 | 60.7 | 60.6 | 60.6 | 1.54 | 0.65 | 41.5 | 100 | 48.9 | NA | 0.59 |
| **≥ 6** | 25.6 | 98.4 | 39.2 | 16.3 | 0.76 | 28.1 | 86.3 | 50.3 | 2.05 | 0.83 | 17.1 | 100 | 27.7 | NA | 0.83 |
| **≥ 7** | 5.83 | 100 | 23.4 | NA | 0.94 | 7.97 | 97.1 | 42.0 | 2.75 | 0.95 | 7.32 | 100 | 19.2 | NA | 0.93 |
| **≥ 8** | 0.45 | 100 | 19.0 | NA | 1.00 | 1.28 | 99.7 | 38.8 | NA | 1.00 | 2.44 | 100 | 14.9 | NA | 0.98 |
| **≥ 9** | 0.00 | 100 | 18.6 | NA | 1.00 | 0.07 | 100 | 38.2 | NA | 1.00 | 0.00 | 100 | 12.8 | NA | 1.00 |
| **>9** | NA | NA | NA | NA | NA | 0.00 | 100 | 38.2 | NA | 1.00 | NA | NA | NA | NA | NA |
| **AUC** | 0.79 (95% CI: 0.77, 0.82) | | | | | 0.64 (95% CI: 0.63, 0.65) | | | | | 0.65 (95% CI: 0.64, 0.65) | | | | |

^1^Values are percentages (except for the AUC values). AUC, area under the curve; LR+; positive likelihood ratio; LR-, negative likelihood ratio; MAR, Mean Adequacy Ratio; NA, not applicable; PCC, percentage correctly classified.

**Supplemental table 8 cont’d. Accuracy of food group cut-offs for an MAR >0.6 among non-pregnant non-lactating adolescent girls (10-19 years), by survey on FAO/ WHO GIFT^1^**

| **Country** | **Italy ‘05 (*n*=162)** | | | | | **Romania ‘12 (*n*=281)** | | | | | **Bulgaria ‘04 (*n*=106)** | | | | |
| --- | --- | --- | --- | --- | --- | --- | --- | --- | --- | --- | --- | --- | --- | --- | --- |
| **Food group cut-off** | Sensitivity | Specificity | PCC | LR+ | LR- | Sensitivity | Specificity | PCC | LR+ | LR- | Sensitivity | Specificity | PCC | LR+ | LR- |
| **≥ 1** | NA | NA | NA | NA | NA | 100 | 0.00 | 92.9 | 1.00 | NA | 100 | 0.00 | 81.1 | 1.00 | NA |
| **≥ 2** | 100 | 0.00 | 98.2 | 1.00 | NA | 100 | 10.0 | 93.6 | 1.11 | 0.00 | 100 | 5.00 | 82.1 | 1.05 | 0.00 |
| **≥ 3** | 99.4 | 0.00 | 97.5 | 0.99 | NA | 99.2 | 40.0 | 95.0 | 1.65 | 0.02 | 97.7 | 15.0 | 82.1 | 1.15 | 0.16 |
| **≥ 4** | 95.6 | 33.3 | 94.4 | 1.43 | 0.13 | 93.1 | 50.0 | 90.0 | 1.86 | 0.14 | 81.4 | 50.0 | 75.5 | 1.63 | 0.37 |
| **≥ 5** | 76.1 | 100 | 76.5 | NA | 0.24 | 80.8 | 80.0 | 80.8 | 4.04 | 0.24 | 39.5 | 75.0 | 46.2 | 1.58 | 0.81 |
| **≥ 6** | 45.3 | 100 | 46.3 | NA | 0.55 | 57.1 | 95.0 | 59.8 | 11.4 | 0.45 | 11.6 | 95.0 | 27.4 | 2.32 | 0.93 |
| **≥ 7** | 8.18 | 100 | 9.88 | NA | 0.92 | 24.9 | 100 | 30.3 | NA | 0.75 | 1.16 | 100 | 19.8 | NA | 0.99 |
| **≥ 8** | 1.26 | 100 | 3.09 | NA | 0.98 | 3.83 | 100 | 10.7 | NA | 0.96 | 0.00 | 100 | 18.9 | NA | 1.00 |
| **≥ 9** | 0.00 | 100 | 1.85 | NA | 1.00 | 0.00 | 100 | 7.12 | NA | 1.00 | NA | NA | NA | NA | NA |
| **>9** | NA | NA | NA | NA | NA | NA | NA | NA | NA | NA | NA | NA | NA | NA | NA |
| **AUC** | 0.90 (95% CI: 0.81, 0.98) | | | | | 0.88 (95% CI: 0.81, 0.95) | | | | | 0.67 (95% CI: 0.53, 0.81) | | | | |

^1^Values are percentages (except for the AUC values). AUC, area under the curve; LR+; positive likelihood ratio; LR-, negative likelihood ratio; MAR, Mean Adequacy Ratio; NA, not applicable; PCC, percentage correctly classified.

**Supplemental table 8 cont’d. Accuracy of food group cut-offs for an MAR >0.6 among non-pregnant non-lactating adolescent girls (10-19 years), by survey on FAO/ WHO GIFT^1^**

| **Country** | **Nigeria ‘11 (*n*=24)** | | | | | **Zambia ‘09 (*n*=4)** | | | | | **Mozambique ‘10 (*n*=428)** | | | | |
| --- | --- | --- | --- | --- | --- | --- | --- | --- | --- | --- | --- | --- | --- | --- | --- |
| **Food group cut-off** | Sensitivity | Specificity | PCC | LR+ | LR- | Sensitivity | Specificity | PCC | LR+ | LR- | Sensitivity | Specificity | PCC | LR+ | LR- |
| **≥ 1** | NA | NA | NA | NA | NA | NA | NA | NA | NA | NA | 100 | 0.00 | 50.0 | 1.00 | NA |
| **≥ 2** | 100 | 0.00 | 91.7 | 1.00 | NA | NA | NA | NA | NA | NA | 100 | 2.80 | 51.4 | 1.03 | 0.00 |
| **≥ 3** | NA | NA | NA | NA | NA | 100 | 0.00 | 75.0 | 1.00 | NA | 97.7 | 20.1 | 58.9 | 1.22 | 0.12 |
| **≥ 4** | 100 | 50.0 | 95.8 | 2.00 | 0.00 | NA | NA | NA | NA | NA | 86.5 | 64.5 | 75.5 | 2.43 | 0.21 |
| **≥ 5** | 77.3 | 100 | 79.2 | NA | 0.23 | 66.7 | 100 | 75.0 | NA | 0.33 | 43.9 | 90.7 | 67.3 | 4.70 | 0.62 |
| **≥ 6** | 22.7 | 100 | 29.2 | NA | 0.77 | NA | NA | NA | NA | NA | 10.3 | 98.6 | 54.4 | 7.33 | 0.91 |
| **≥ 7** | 4.55 | 100 | 12.5 | NA | 0.95 | 33.3 | 100 | 50.0 | NA | 0.67 | 0.93 | 100 | 50.5 | NA | 0.99 |
| **≥ 8** | 0.00 | 100 | 8.33 | NA | 1.00 | 0.00 | 100 | 25.0 | NA | 1.00 | 0.47 | 100 | 50.2 | NA | 1.00 |
| **≥ 9** | 100 | 0.00 | 91.7 | 1.00 | NA | NA | NA | NA | NA | NA | 0.00 | 100 | 50.0 | NA | 1.00 |
| **>9** | NA | NA | NA | NA | NA | NA | NA | NA | NA | NA | NA | NA | NA | NA | NA |
| **AUC** | 0.94 (95% CI: 0.82, 1.00) | | | | | 0.83 (95% CI: NA) | | | | | 0.80 (95% CI: 0.76, 0.84) | | | | |

^1^Values are percentages (except for the AUC values). AUC, area under the curve; LR+; positive likelihood ratio; LR-, negative likelihood ratio; MAR, Mean Adequacy Ratio; NA, not applicable; PCC, percentage correctly classified.

**Supplemental table 8 cont’d. Accuracy of food group cut-offs for an MAR >0.6 among non-pregnant non-lactating adolescent girls (10-19 years), by survey on FAO/ WHO GIFT^1^**

| **Country** | **Ethiopia ‘13 (South) (*n*=4)** | | | | | **Kenya ‘14 (lean) (*n*=6)** | | | | | **Kenya ‘15 (baseline) (*n*=5)** | | | | |
| --- | --- | --- | --- | --- | --- | --- | --- | --- | --- | --- | --- | --- | --- | --- | --- |
| **Food group cut-off** | Sensitivity | Specificity | PCC | LR+ | LR- | Sensitivity | Specificity | PCC | LR+ | LR- | Sensitivity | Specificity | PCC | LR+ | LR- |
| **≥ 1** | NA | NA | NA | NA | NA | NA | NA | NA | NA | NA | NA | NA | NA | NA | NA |
| **≥ 2** | 100 | 0.00 | 75.00 | 1.00 | NA | NA | NA | NA | NA | NA | 100 | 0.00 | 80.0 | 1.00 | NA |
| **≥ 3** | 100 | 100 | 100 | NA | 0.00 | NA | NA | NA | NA | NA | 100 | 100 | 100 | NA | 0.00 |
| **≥ 4** | NA | NA | NA | NA | NA | 100 | 0.00 | 83.3 | 1.00 | NA | 75.0 | 100 | 80.0 | NA | 0.25 |
| **≥ 5** | 33.3 | 0.00 | 25.0 | 0.33 | NA | 60.0 | 0.00 | 50.0 | 0.60 | NA | 25.0 | 100 | 40.0 | NA | 0.75 |
| **≥ 6** | 0.00 | 100 | 25.0 | NA | 1.00 | 0.00 | 100 | 16.7 | NA | 1.00 | 0.00 | 100 | 20.0 | NA | 1.00 |
| **≥ 7** | NA | NA | NA | NA | NA | NA | NA | NA | NA | NA | NA | NA | NA | NA | NA |
| **≥ 8** | NA | NA | NA | NA | NA | NA | NA | NA | NA | NA | NA | NA | NA | NA | NA |
| **≥ 9** | NA | NA | NA | NA | NA | NA | NA | NA | NA | NA | NA | NA | NA | NA | NA |
| **>9** | NA | NA | NA | NA | NA | NA | NA | NA | NA | NA | NA | NA | NA | NA | NA |
| **AUC** | 0.00 (95% CI: NA) | | | | | 0.30 (95% CI: NA) | | | | | 1.00 (95% CI: NA) | | | | |

^1^Values are percentages (except for the AUC values). AUC, area under the curve; LR+; positive likelihood ratio; LR-, negative likelihood ratio; MAR, Mean Adequacy Ratio; NA, not applicable; PCC, percentage correctly classified.

**Supplemental table 8 cont’d. Accuracy of food group cut-offs for an MAR >0.6 among non-pregnant non-lactating adolescent girls (10-19 years), by survey on FAO/ WHO GIFT^1^**

| **Country** | **Kenya ‘16 (*n*=18)** | | | | | **Kenya ‘18 (*n*=14)** | | | | | **India ‘09 (*n*=3,827)** | | | | |
| --- | --- | --- | --- | --- | --- | --- | --- | --- | --- | --- | --- | --- | --- | --- | --- |
| **Food group cut-off** | Sensitivity | Specificity | PCC | LR+ | LR- | Sensitivity | Specificity | PCC | LR+ | LR- | Sensitivity | Specificity | PCC | LR+ | LR- |
| **≥ 1** | NA | NA | NA | NA | NA | 100 | 0.00 | 57.1 | 1.00 | NA | 100 | 0.00 | 44.2 | 1.00 | NA |
| **≥ 2** | 100 | 0.00 | 77.8 | 1.00 | NA | 100 | 16.7 | 64.3 | 1.20 | 0.00 | 99.5 | 3.19 | 45.8 | 1.03 | 0.17 |
| **≥ 3** | 78.6 | 25.0 | 66.7 | 1.05 | 0.86 | 87.5 | 33.3 | 64.3 | 1.31 | 0.38 | 88.2 | 23.0 | 51.8 | 1.14 | 0.52 |
| **≥ 4** | 64.3 | 100 | 72.2 | NA | 0.36 | 50.0 | 50.0 | 50.0 | 1.00 | 1.00 | 53.5 | 63.0 | 58.8 | 1.44 | 0.74 |
| **≥ 5** | 28.6 | 100 | 44.4 | NA | 0.71 | 50.0 | 100 | 71.4 | NA | 0.50 | 17.1 | 91.1 | 58.4 | 1.92 | 0.91 |
| **≥ 6** | 7.14 | 100 | 27.8 | NA | 0.93 | 12.5 | 100 | 50.0 | NA | 0.88 | 4.49 | 98.4 | 56.9 | 2.74 | 0.97 |
| **≥ 7** | 0.00 | 100 | 22.2 | NA | 1.00 | 0.00 | 100 | 42.9 | NA | 1.00 | 1.00 | 99.9 | 56.2 | 10.7 | 0.99 |
| **≥ 8** | NA | NA | NA | NA | NA | NA | NA | NA | NA | NA | 0.00 | 100 | 55.8 | NA | 1.00 |
| **≥ 9** | NA | NA | NA | NA | NA | NA | NA | NA | NA | NA | 0.00 | 100 | 50.0 | NA | 1.00 |
| **>9** | NA | NA | NA | NA | NA | NA | NA | NA | NA | NA | NA | NA | NA | NA | NA |
| **AUC** | 0.76 (95% CI: 0.55, 0.97) | | | | | 0.69 (95% CI: 0.39, 0.99) | | | | | 0.61 (95% CI: 0.59, 0.63) | | | | |

^1^Values are percentages (except for the AUC values). AUC, area under the curve; LR+; positive likelihood ratio; LR-, negative likelihood ratio; MAR, Mean Adequacy Ratio; NA, not applicable; PCC, percentage correctly classified.

**Supplemental table 8 cont’d. Accuracy of food group cut-offs for an MAR >0.6 among non-pregnant non-lactating adolescent girls (10-19 years), by survey on FAO/ WHO GIFT^1^**

| **Country** | **Bangladesh ‘07 (*n*=5)** | | | | | **Lao PDR ‘16 (*n*=98)** | | | | |
| --- | --- | --- | --- | --- | --- | --- | --- | --- | --- | --- |
| **Food group cut-off** | Sensitivity | Specificity | PCC | LR+ | LR- | Sensitivity | Specificity | PCC | LR+ | LR- |
| **≥ 1** | NA | NA | NA | NA | NA | NA | NA | NA | NA | NA |
| **≥ 2** | 100 | 0.00 | 80.0 | 1.00 | NA | 100 | 0.00 | 32.7 | 1.00 | NA |
| **≥ 3** | 75.0 | 0.00 | 60.0 | 0.75 | NA | 96.9 | 7.58 | 36.7 | 1.05 | 0.41 |
| **≥ 4** | NA | NA | NA | NA | NA | 78.1 | 48.5 | 58.2 | 1.52 | 0.45 |
| **≥ 5** | 50.0 | 100 | 60.0 | NA | 0.50 | 46.9 | 87.9 | 74.5 | 3.87 | 0.60 |
| **≥ 6** | 25.0 | 100 | 40.0 | NA | 0.75 | 18.8 | 98.5 | 72.5 | 12.4 | 0.83 |
| **≥ 7** | 0.00 | 100 | 100 | NA | 1.00 | 3.13 | 98.5 | 67.4 | 2.06 | 0.98 |
| **≥ 8** | NA | NA | NA | NA | NA | 0.00 | 100 | 67.4 | NA | 1.00 |
| **≥ 9** | NA | NA | NA | NA | NA | NA | NA | NA | NA | NA |
| **>9** | NA | NA | NA | NA | NA | NA | NA | NA | NA | NA |
| **AUC** | 0.62 (95% CI: NA) | | | | | 0.71 (95% CI: 0.60, 0.82) | | | | |

^1^Values are percentages (except for the AUC values). AUC, area under the curve; LR+; positive likelihood ratio; LR-, negative likelihood ratio; MAR, Mean Adequacy Ratio; NA, not applicable; PCC, percentage correctly classified; PDR, People’s Democratic Republic.

|  | **Mexico ‘12** | **Brazil ‘13** | **Italy ‘05** | **Total** |
| --- | --- | --- | --- | --- |
| **Calcium (PA)** | 0.72 ± 0.15 | 0.00 ± 0.00 | 0.78 ± 0.11 | 0.03 ± 0.16 |
| **Iron (PA)** | 1.00 ± 0.00 | 1.00 ± 0.00 | 1.00 ± 0.00 | 1.00 ± 0.00 |
| **Zinc (PA)** | 1.00 ± 0.00 | 1.00 ± 0.00 | **NA** | 1.00 ± 0.00^2^ |
| **Vitamin A (PA)** | 0.30 ± 0.28 | 0.00 ± 0.00 | 1.00 ± 0.00 | 0.02 ± 0.10 |
| **Thiamin (PA)** | 1.00 ± 0.00 | 1.00 ± 0.00 | 1.00 ± 0.00 | 1.00 ± 0.00 |
| **Riboflavin (PA)** | 1.00 ± 0.00 | 1.00 ± 0.00 | 1.00 ± 0.00 | 1.00 ± 0.00 |
| **Niacin (PA)** | 1.00 ± 0.00 | 1.00 ± 0.00 | 1.00 ± 0.00 | 1.00 ± 0.00 |
| **Vitamin B6 (PA)** | 1.00 ± 0.00 | 0.33 ± 0.31 | 1.00 ± 0.00 | 0.36 ± 0.33 |
| **Folate (PA)** | 1.00 ± 0.00 | 1.00 ± 0.00 | 1.00 ± 0.00 | 1.00 ± 0.00 |
| **Vitamin B12 (PA)** | 1.00 ± 0.00 | 1.00 ± 0.00 | 1.00 ± 0.00 | 1.00 ± 0.00 |
| **Vitamin C (PA)** | 0.59 ± 0.46 | 0.11 ± 0.12 | 1.00 ± 0.00 | 0.13 ± 0.19 |
| **MPA** | 0.87 ± 0.07 | 0.68 ± 0.04 | 0.98 ± 0.01 | 0.69 ± 0.06 |
| **MPA >0.60** | 1,411 (100) | 32,059 (100) | 139 (100) | 33,609 (100) |
| **Sample size, 1-d 24-HR** | 1,411 | 32,059 | 139 | 33,609 |
| **Sample size, 2-d 24-HR** | 121 | 2,179 | 139 | 2,439 |
| **Sample size, 3-d 24-HR** | NA | NA | 139 | 139 |

**Supplemental table 9. PAs of 11 micronutrients and MPA among adolescent boys (10-19 years), by survey on FAO/ WHO GIFT^1^**

^1^Data are mean ± SD or frequency (%). 24-HR, 24-hour recall; GIFT, Global Individual Food consumption data Tool; NA, not applicable; MPA, Mean Probability of Adequacy; PA, Probability of Adequacy.

^2^Excluding Italy, thus *n*=33,470.

|  | **Mexico ‘12** | **Brazil ‘13** | **Italy ‘05** | **Mozambique ‘10** | **Total** |
| --- | --- | --- | --- | --- | --- |
| **Calcium (PA)** | 0.46 ± 0.22 | 0.00 ± 0.00 | 0.25 ± 0.26 | 0.00 ± 0.00 | 0.02 ± 0.09 |
| **Iron (PA)** | 1.00 ± 0.00 | 1.00 ± 0.00 | 1.00 ± 0.00 | 0.12 ± 0.09 | 0.99 ± 0.09 |
| **Zinc (PA)** | 1.00 ± 0.00 | 0.28 ± 0.15 | **NA** | 0.28 ± 0.15 | 0.98 ± 0.07^2^ |
| **Vitamin A (PA)** | 0.25 ± 0.22 | 0.00 ± 0.00 | 1.00 ± 0.00 | 0.00 ± 0.00 | 0.09 ± 0.01 |
| **Thiamin (PA)** | 1.00 ± 0.00 | 1.00 ± 0.00 | 1.00 ± 0.00 | 1.00 ± 0.00 | 1.00 ± 0.00 |
| **Riboflavin (PA)** | 1.00 ± 0.00 | 0.98 ± 0.02 | 1.00 ± 0.00 | 0.03 ± 0.08 | 0.97 ± 0.10 |
| **Niacin (PA)** | 1.00 ± 0.00 | 1.00 ± 0.00 | 1.00 ± 0.00 | 0.79 ± 0.09 | 0.99 ± 0.02 |
| **Vitamin B6 (PA)** | 1.00 ± 0.00 | 0.10 ± 0.08 | 1.00 ± 0.00 | 1.00 ± 0.00 | 0.14 ± 0.21 |
| **Folate (PA)** | 1.00 ± 0.00 | 1.00 ± 0.00 | 1.00 ± 0.00 | 0.62 ± 0.14 | 1.00 ± 0.04 |
| **Vitamin B12 (PA)** | 1.00 ± 0.00 | 1.00 ± 0.00 | 1.00 ± 0.00 | 0.78 ± 0.11 | 1.00 ± 0.02 |
| **Vitamin C (PA)** | 0.69 ± 0.34 | 0.09 ± 0.09 | 1.00 ± 0.00 | 1.00 ± 0.00 | 0.12 ± 0.19 |
| **MPA** | 0.85 ± 0.05 | 0.65 ± 0.02 | 0.93 ± 0.03 | 0.51 ± 0.06 | 0.66 ± 0.05 |
| **MPA >0.60** | 1,369 (100) | 39,912 (100) | 162 (100) | 85 (19.9) | 41,528 (99.2) |
| **Sample size, 1-d 24-HR** | 1,369 | 39,912 | 162 | 428 | 41,871 |
| **Sample size, 2-d 24-HR** | 123 | 2,807 | 162 | 87 | 3,179 |
| **Sample size, 3-d 24-HR** | **NA** | **NA** | 162 | 76 | 238 |
| **Sample size, 4-d 24-HR** | **NA** | **NA** | **NA** | 67 | 67 |

**Supplemental table 10. PAs of 11 micronutrients and MPA among non-pregnant non-lactating adolescent girls (10-19 years), by survey on FAO/ WHO GIFT^1^**

^1^Data are mean ± SD or frequency (%). 24-HR, 24-hour recall; GIFT, Global Individual Food consumption data Tool; NA, not applicable; MPA, Mean Probability of Adequacy; PA, Probability of Adequacy.

^2^Excluding Italy, thus *n*=41,366.


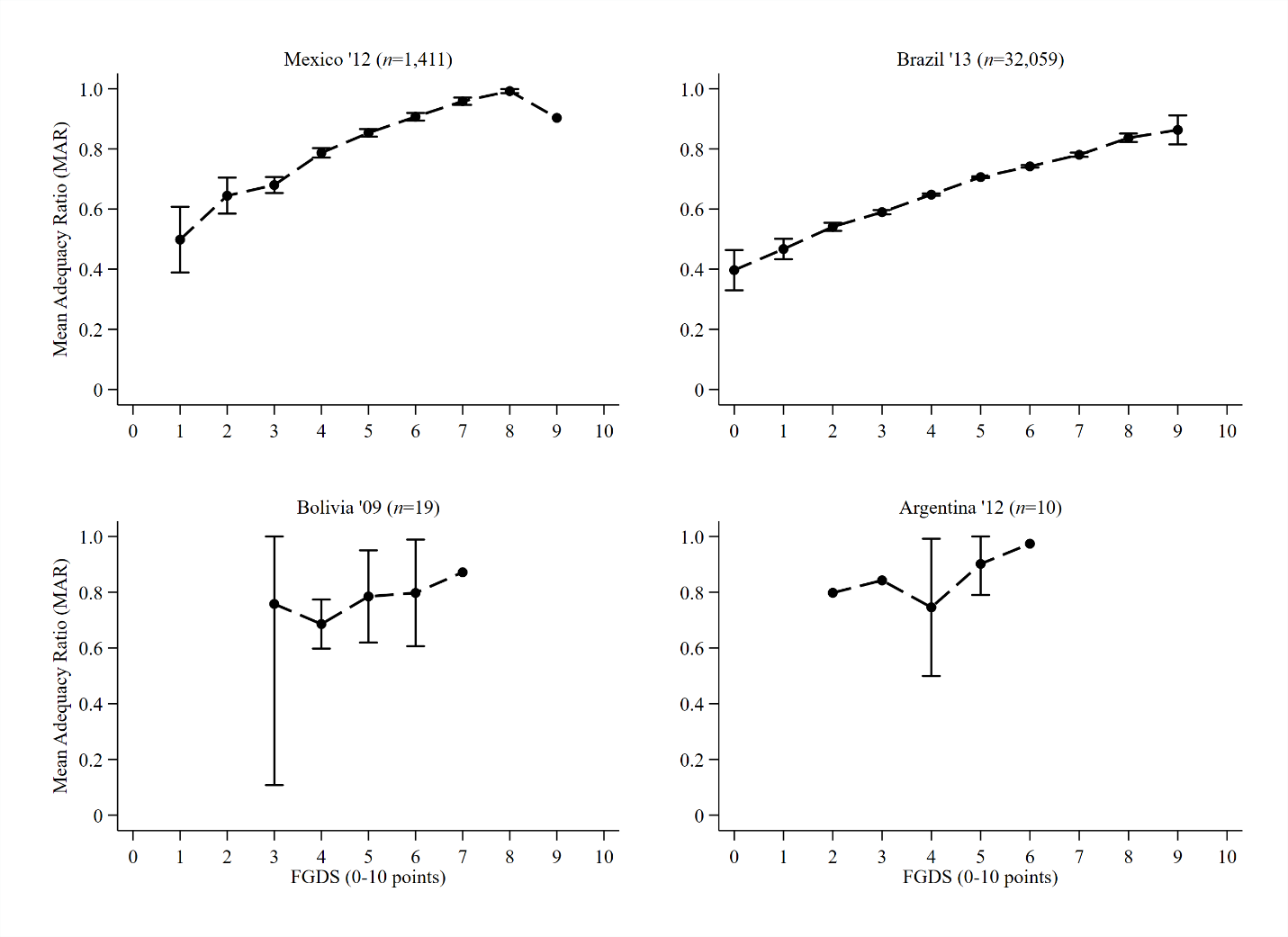
**Supplemental figure 1. Relationships between the MAR of 11 micronutrients and food group diversity score (FGDS) among adolescent boys (10-19 years) from Mexico, Brazil, Bolivia, and Argentina.** Error bars represent the 95% confidence intervals around the means.

**
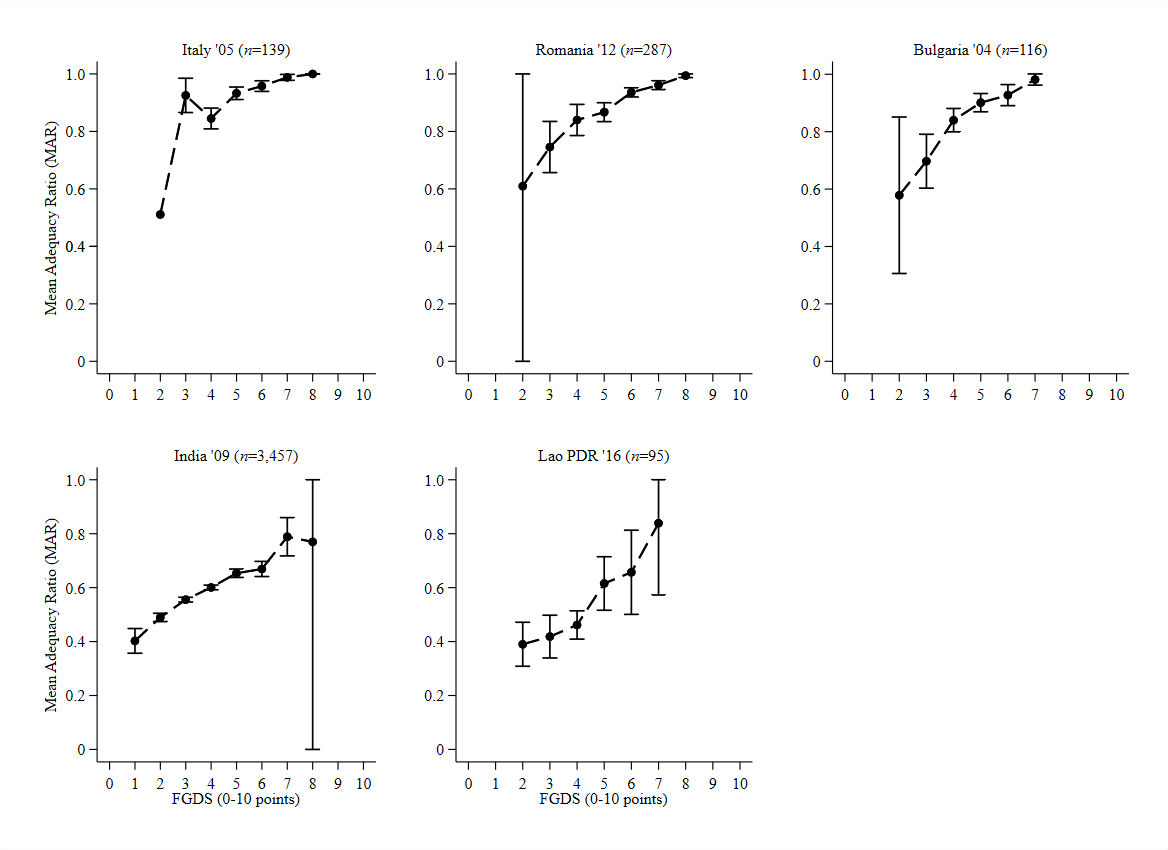
Supplemental figure 2. Relationships between the MAR of 11 micronutrients and food group diversity score (FGDS) among adolescent boys (10-19 years) from Italy, Romania, Bulgaria, India, and Lao PDR.** Error bars represent the 95% confidence intervals around the means. MAR was calculated for 10, 9, and 7 micronutrients in Italy (zinc missing), India (vitamin B6 and vitamin B12 missing), and Lao PDR (zinc, vitamin B6, folate, and vitamin B12 missing), respectively. PDR, People’s Democratic Republic.

**
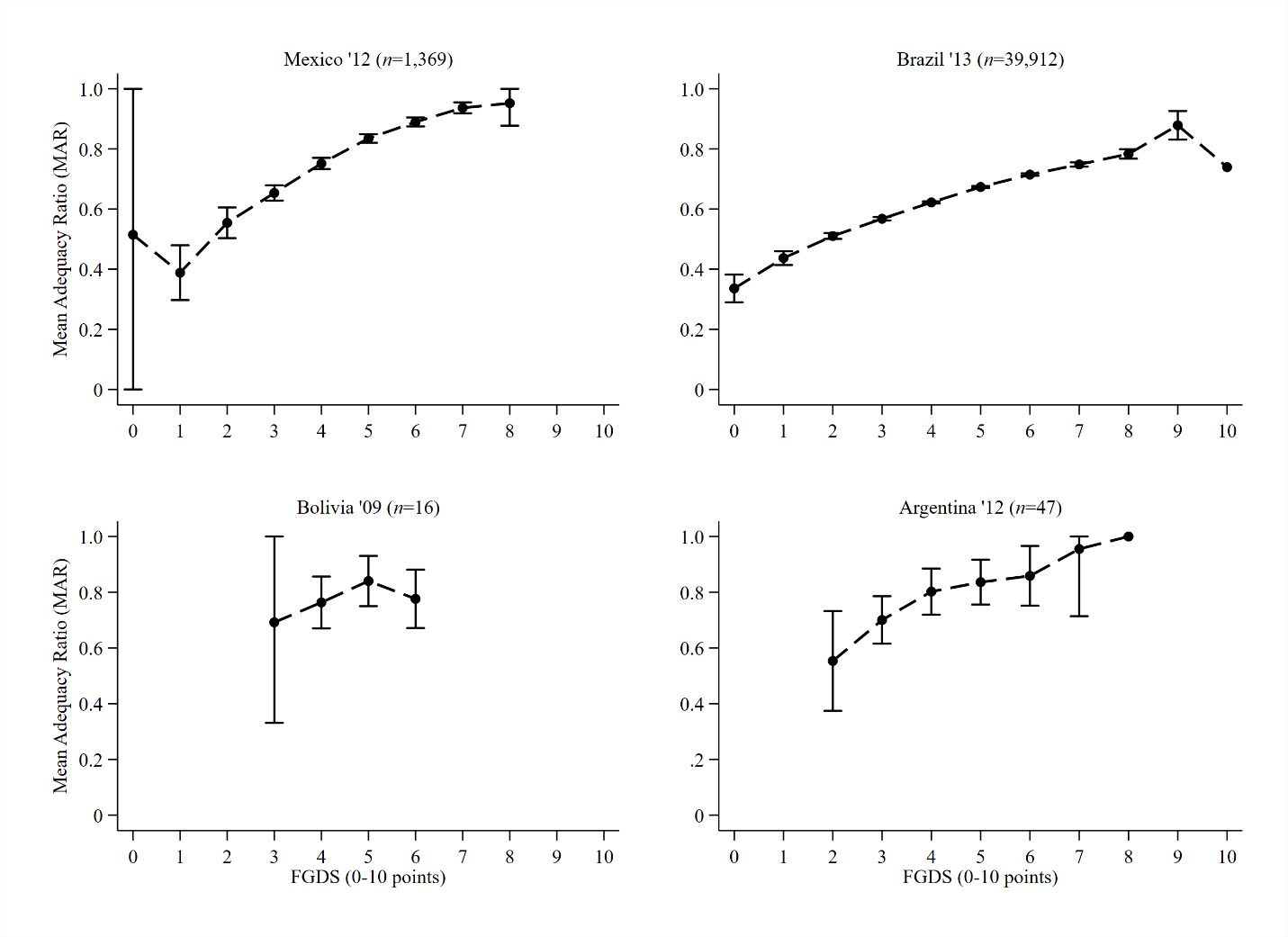
Supplemental figure 3. Relationships between the MAR of 11 micronutrients and food group diversity score (FGDS) among non-pregnant non-lactating adolescent girls (10-19 years) from Mexico, Brazil, Bolivia, and Argentina.** Error bars represent the 95% confidence intervals around the means.

**
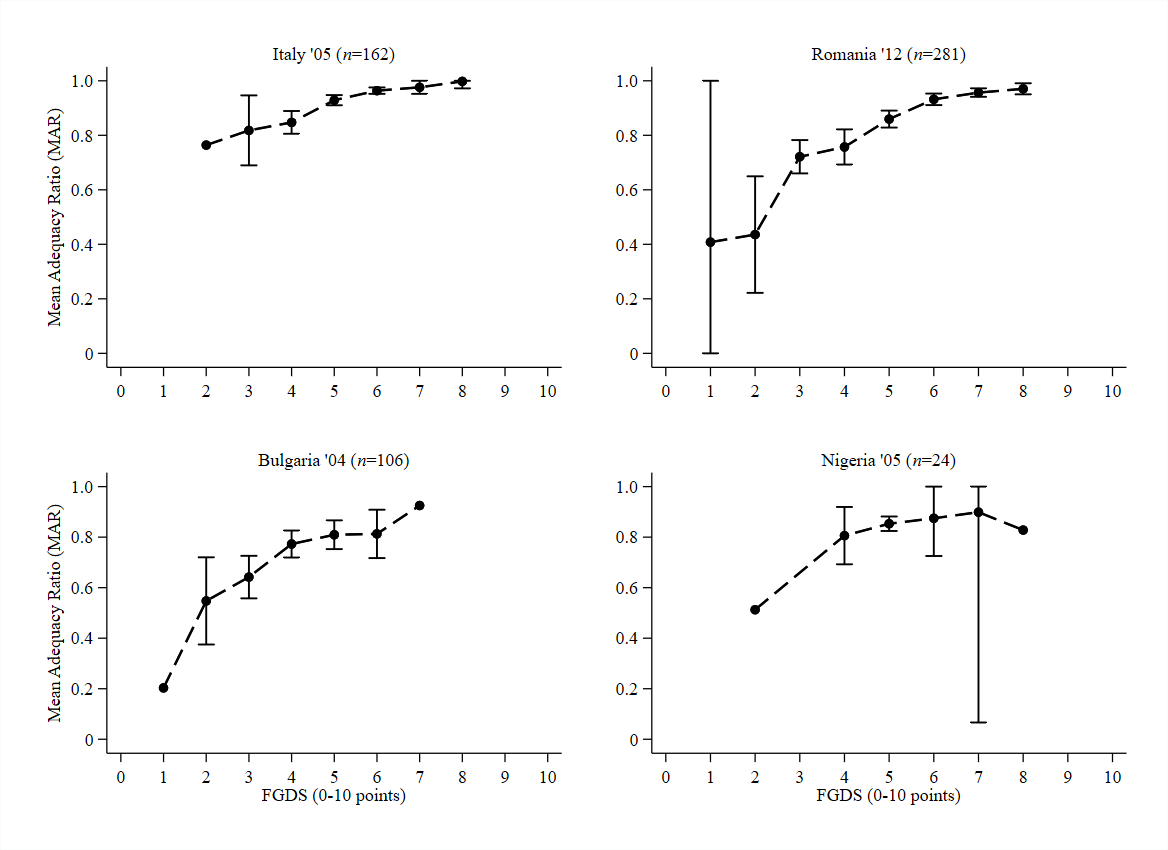
Supplemental figure 4. Relationships between the MAR of 11 micronutrients and food group diversity score (FGDS) among non-pregnant non-lactating adolescent girls (10-19 years) from Italy, Romania, Bulgaria, and Nigeria.** Error bars represent the 95% confidence intervals around the means. MAR was calculated for 10 micronutrients in Italy (zinc missing).

**
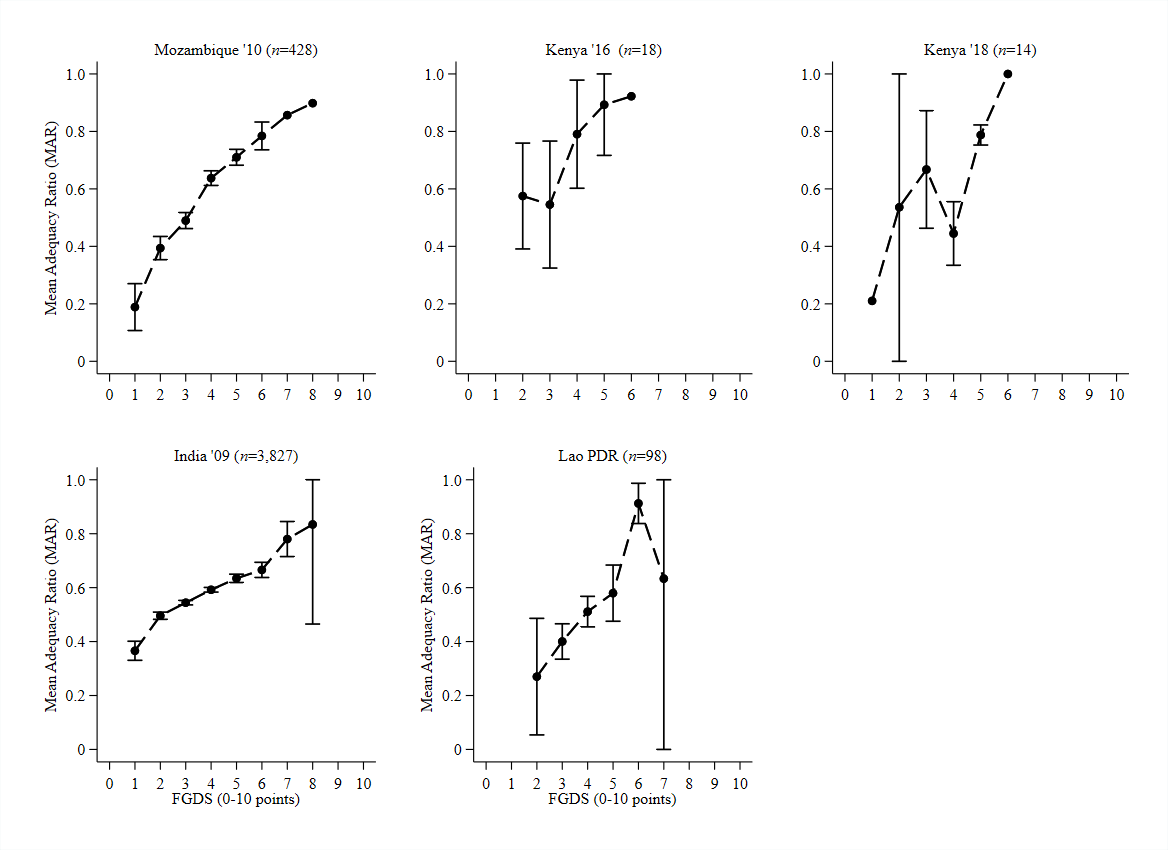
Supplemental figure 5. Relationships between the MAR of 11 micronutrients and food group diversity score (FGDS) among non-pregnant non-lactating adolescent girls (10-19 years) from Mozambique, Kenya, India, and Lao PDR.** Error bars represent the 95% confidence intervals around the means. MAR was calculated for 10, 9, and 7 micronutrients in Kenya ’18 (vitamin B6 missing), India (vitamin B6 and vitamin B12 missing) and Lao PDR (zinc, vitamin B6, folate, and vitamin B12 missing), respectively. PDR, People’s Democratic Republic.

**
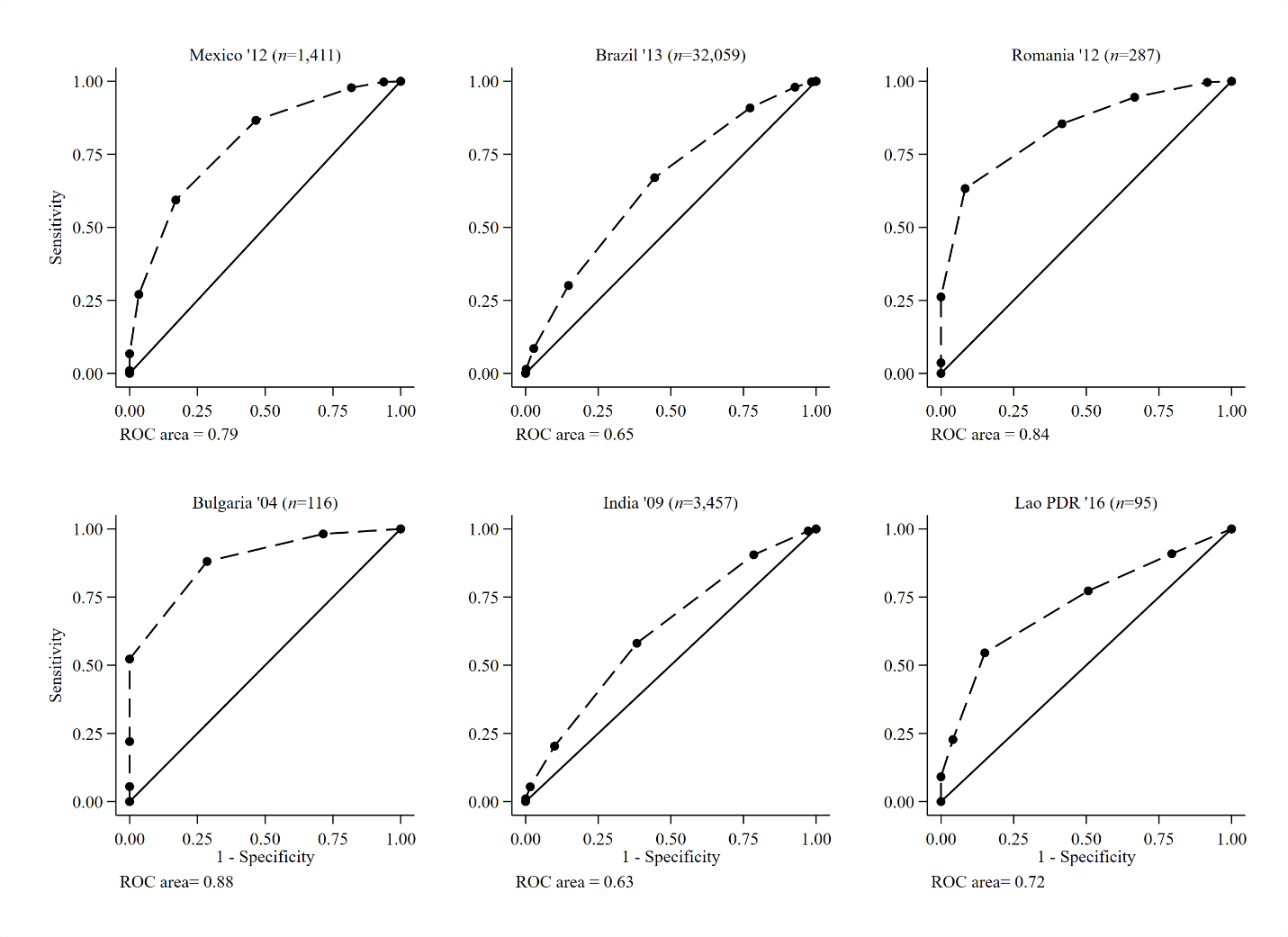
Supplemental figure 6**. **Receiver operating characteristic (ROC) curves of the food group diversity score (0-10 points) underlying MDD-W indicating predictions for adolescent boys (10-19 years) MAR >0.60 for 11 micronutrients.** MAR was calculated for 9 and 7 micronutrients in India (vitamin B6 and vitamin B12 missing) and Lao PDR (zinc, vitamin B6, folate, and vitamin B12 missing), respectively. MDD-W; Minimum Dietary Diversity for Women; PDR, People’s Democratic Republic.

**
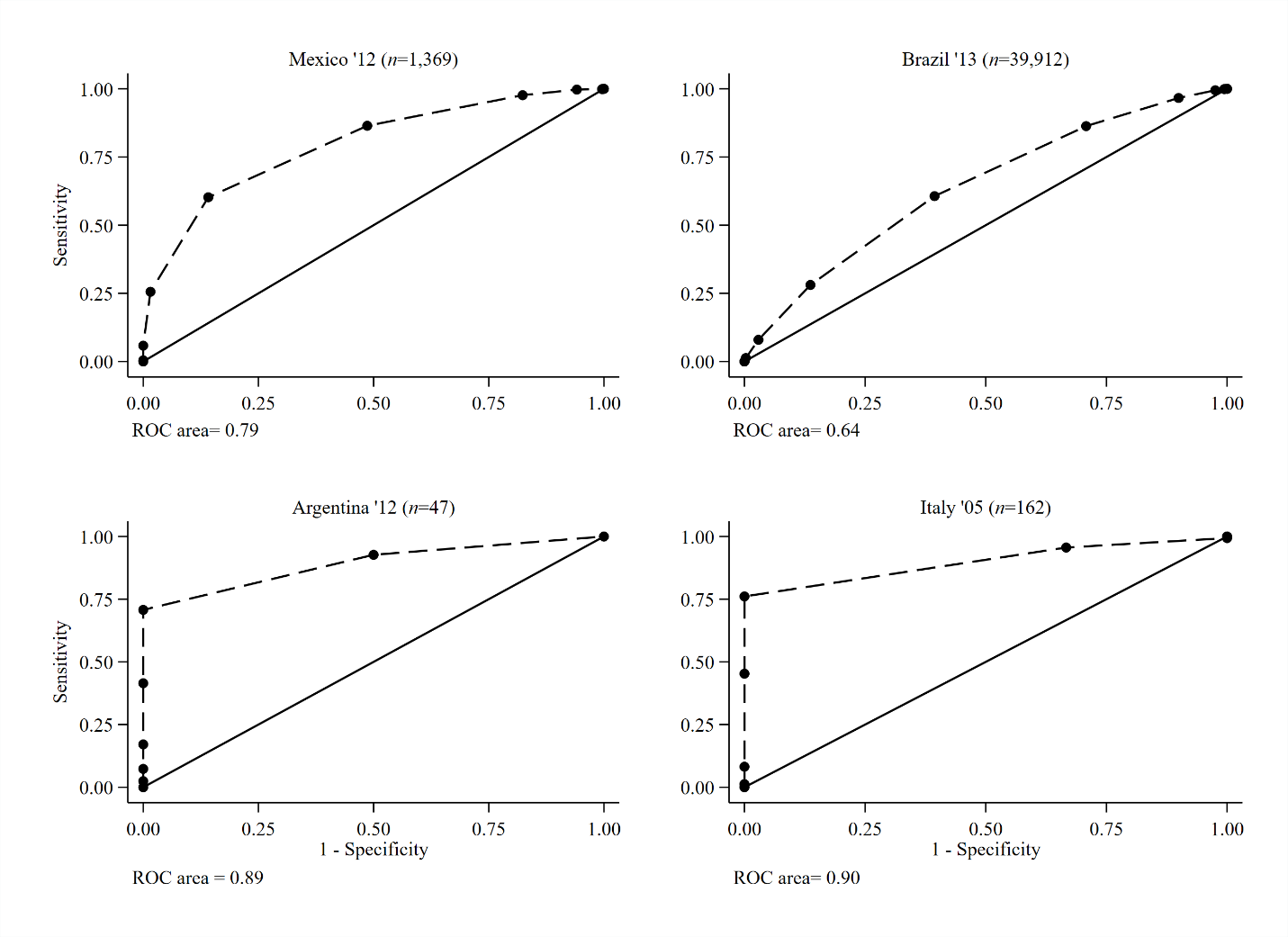
Supplemental figure 7**. **Receiver operating characteristic (ROC) curves of the food group diversity score (0-10 points) underlying MDD-W indicating predictions for non-pregnant non-lactating adolescent girls (10-19 years) MAR >0.60 for 11 micronutrients in Mexico, Brazil, Argentina, and Italy.** MAR was calculated for 10 micronutrients in Italy (zinc missing). MAR, Mean Adequacy Ratio; MDD-W, Minimum Dietary Diversity for Women.

**
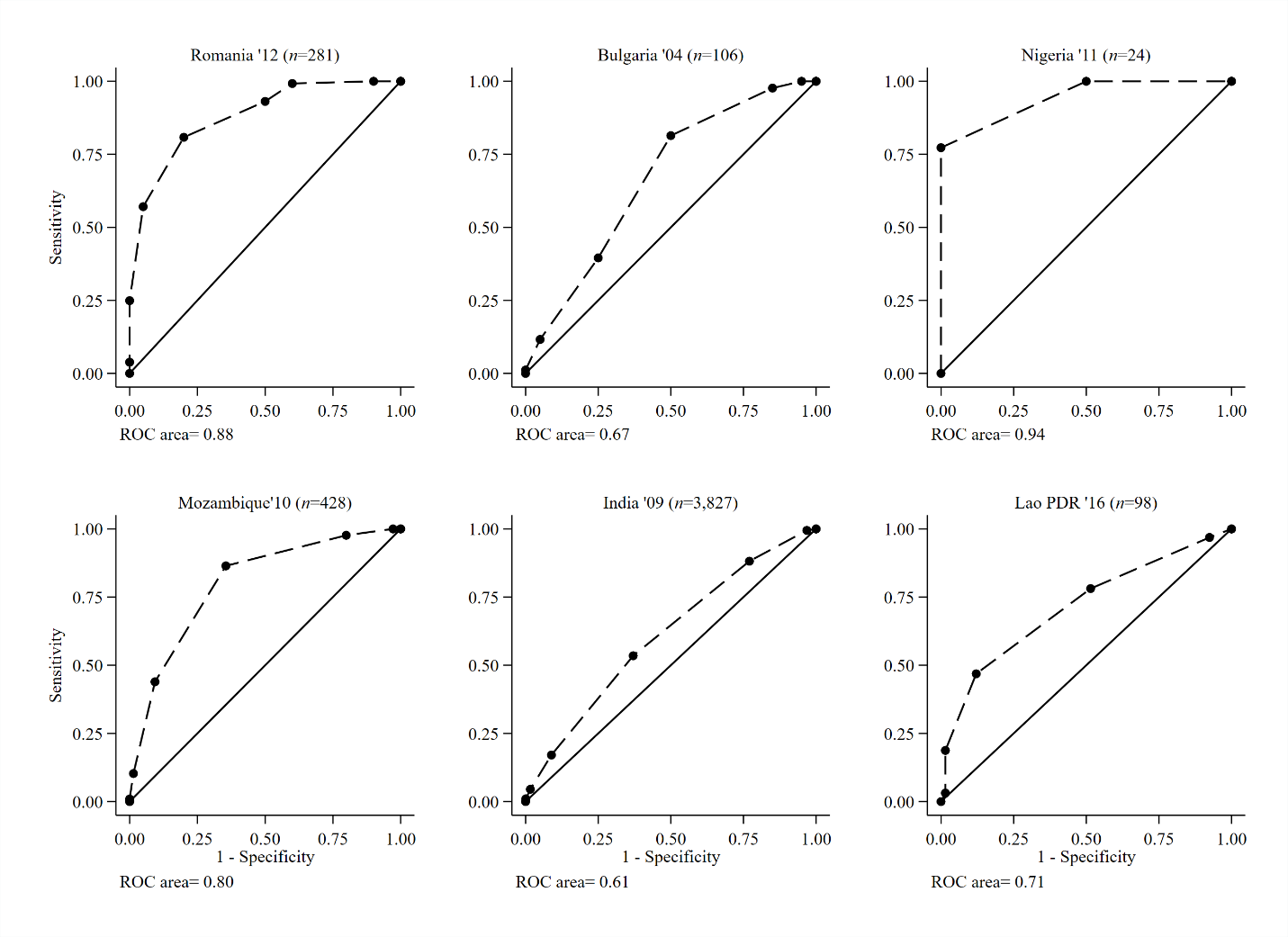
Supplemental figure 8**. **Receiver operating characteristic (ROC) curves of the food group diversity score (0-10 points) underlying MDD-W indicating predictions for non-pregnant non-lactating adolescent girls (10-19 years) MAR >0.60 for 11 micronutrients in Romania, Bulgaria, Nigeria, Mozambique, India, and Lao PDR.** MAR was calculated for 9 and 7 micronutrients in India (vitamin B6 and vitamin B12 missing) and Lao PDR (zinc, vitamin B6, folate, and vitamin B12 missing), respectively. MAR, Mean Adequacy Ratio; MDD-W, Minimum Dietary Diversity for Women; PDR, People’s Democratic Republic.
